# Semi-Synthesis and Evaluation of Sargahydroquinoic Acid Derivatives as Potential Antimalarial Agents

**DOI:** 10.3390/medicines6020047

**Published:** 2019-04-01

**Authors:** Tatenda C. Munedzimwe, Robyn L. van Zyl, Donovan C. Heslop, Adrienne L. Edkins, Denzil R. Beukes

**Affiliations:** 1 Faculty of Pharmacy, Rhodes University, Grahamstown 6139, South Africa; tatendamunedzimwe@gmail.com; 2 Pharmacology Division, Department of Pharmacy and Pharmacology, WITS Research Institute for Malaria (WRIM), MRC Collaborating Centre for Multidisciplinary Research on Malaria, Faculty of Health Sciences, University of the Witwatersrand, Johannesburg 2000, South Africa; robyn.vanzyl@wits.ac.za (R.L.v.Z.); donoheslop@gmail.com (D.C.H.); 3 Biomedical Biotechnology Research Unit (BioBRU), Department of Biochemistry and Microbiology, Rhodes University, Grahamstown 6139, South Africa; a.edkins@ru.ac.za; 4 School of Pharmacy, University of the Western Cape, Bellville 7535, South Africa

**Keywords:** sargaquinoic acid, sarganaphthoquinoic acid, antiplasmodial, malaria

## Abstract

**Background:** Malaria continues to present a major health problem, especially in developing countries. The development of new antimalarial drugs to counter drug resistance and ensure a steady supply of new treatment options is therefore an important area of research. Meroditerpenes have previously been shown to exhibit antiplasmodial activity against a chloroquinone sensitive strain of *Plasmodium falciparum* (D10). In this study we explored the antiplasmodial activity of several semi-synthetic analogs of sargahydroquinoic acid. **Methods:** Sargahydroquinoic acid was isolated from the marine brown alga, *Sargassum incisifolium* and converted, semi-synthetically, to several analogs. The natural products, together with their synthetic derivatives were evaluated for their activity against the FCR-3 strain of *Plasmodium falciparum* as well as MDA-MB-231 breast cancer cells. **Results:** Sarganaphthoquinoic acid and sargaquinoic acid showed the most promising antiplasmodial activity and low cytotoxicity. **Conclusions:** Synthetic modification of the natural product, sargahydroquinoic acid, resulted in the discovery of a highly selective antiplasmodial compound, sarganaphthoquinoic acid.

## 1. Introduction

**Figure 1:**
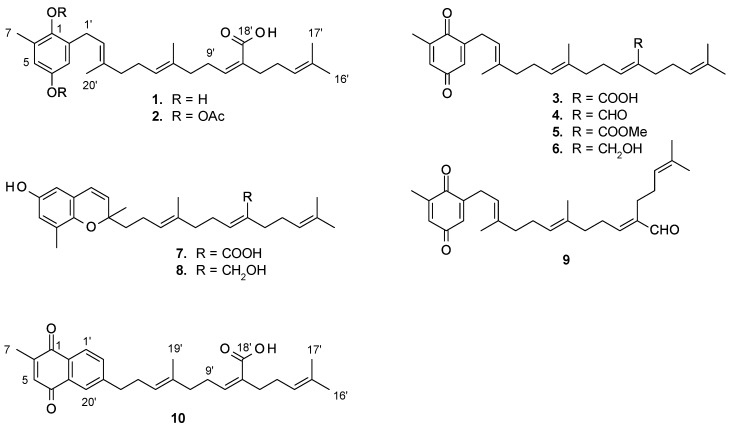
Natural and semi-synthetic derivatives of sargahydroquinoic acid (**1**).

Despite the impressive breakthroughs in the treatment of malaria [1], it remains a life-threatening disease. Southeast Asia and sub-Saharan Africa account for the vast majority of the estimated 219 million malaria cases reported worldwide, leading to 435,000 deaths [2]. More than 90% of malaria cases and deaths occur in Africa, of which more than 70% are children under five years of age [2]. The prospect of resistance to current drugs appears inevitable and paints a bleak picture indeed [3]. Although the reasons for this dire situation are complex, there is undoubtedly a need for the continued search for and development of new antimalarial drugs. Natural products have historically offered some of the most effective antimalarial drugs [4]. In a previous study, we reported on the antiplasmodial activity of natural products isolated from the South African brown seaweed, *Sargassum incisifolium,* against a chloroquine sensitive strain of *Plasmodium falciparum* (D10) [5]. *S. incisifolium* is relatively abundant along the South African coastline and produces sargahydroquinoic acid (**1**) as the major metabolite. The accessibility of **1** thus provided an opportunity to explore the structure activity relationships of analogs of this natural product. Herein we report on the antiplasmodial activity of semi-synthetic analogs of sargahydroquinoic acid (**1**) (Figure 1). 

## 2. Materials and Methods 

### 2.1. General Experimental 

All solvents were of chromatographic grade (Merck, Darmstadt, Germany) and used without further purification. Column chromatography was performed on silica gel (40–63 μm particle size) from Merck, Darmstadt, Germany. Normal Phase HPLC was carried out using a Whatman Partisil 10 semi-preparative column (Sigma-Aldrich, Schnelldorf, Germany) (10 mm × 500 mm, 10 μm), while a Phenomenex Luna C_18_ column (Sigma-Aldrich, Schnelldorf, Germany, 10 mm × 250 mm, 10 μm) was used for reversed phase HPLC. NMR spectra were recorded on Bruker Avance 400 and 600 MHz spectrometers (Bruker Biospin, Rheinstetten, Germany) and referenced to residual undeuterated CDCl_3_ solvent signals (δ_H_ 7.26 ppm and δ_C_ 77.0 ppm). UV spectra were measured on a Perkin Elmer Lambda 25 UV/Vis spectrometer (Perkin-Elmer, Norwalk, CT, USA) while FT-IR data was obtained using a Perkin Elmer Spectrum 100 FT-IR spectrometer (Perkin-Elmer, Norwalk, CT, USA). High resolution electrospray ionization mass spectroscopy (HR-ESIMS) spectra were obtained on a Waters Synapt G2 mass spectrometer (Waters Corporation, Milford, MA, USA) at 20 V.

### 2.2. Extraction and Isolation of Natural Products

Specimens of *Sargassum incisifolium* were collected from Port Alfred (collection code PA071b) on the south east coast of South Africa on 21 September 2007 and stored at −20 °C. The samples were authenticated by comparison with voucher specimens from previous studies [5]. Voucher specimens are stored at the School of Pharmacy, University of the Western Cape.

The following isolation protocol is representative and was repeated several times in order to generate sufficient quantities of **1** for synthetic modification. The frozen alga (38.77 g, extracted dry weight) was allowed to thaw at room temperature after which it was soaked in methanol for one hour. The methanol was removed and the alga extracted three times with MeOH-CH_2_Cl_2_ (1:2) at 40 °C for 30 min. Extracts were pooled and separated into aqueous and organic phases by the addition of distilled water. Concentration of the organic phase under reduced pressure gave a dark green residue (3.87 g). A portion of the organic fraction (1.09 g) was fractionated by step-gradient elution on a silica gel column (10 g) using solvents of increasing polarity (*n*-hexane-EtOAc) to give seven fractions as follows: Fr A (H-E, 10:0, 8.6 mg), Fr B (H-E, 9:1, 27 mg), Fr C (H-E, 8:2, 132 mg), Fr D (H-E, 6:4, 218 mg), Fr E (H-E, 4:6, 65 mg), Fr F (H-E, 2:8, 9.7 mg) and Fr G (H-E, 0:10, 50 mg) followed by MeOH-EtOAc (1:1), Fr 7H (238 mg). Fraction B (19 mg) was further purified by silica gel column chromatography using a mobile phase of *n*-hexane-EtOAc (9:1) to give 1.7 mg of sargaquinal (**9**). Fraction C (40 mg) was purified by normal phase HPLC using *n*-hexane-EtOAc as mobile phase (8:2) to give 15 mg of sargaquinoic acid (**3**). Fraction D (20 mg) was purified by reversed phase HPLC using MeOH-H_2_O phase (90:10) as the mobile phase to give sargahydroquinoic acid (**1**) (6.8 mg) and sargachromenol (**7**) (2.4 mg), respectively. The isolation of compounds **1**, **3**, **7** and **9** is summarised in Scheme S1 and their structures were confirmed by spectroscopic methods, which were in agreement with literature data (Table S1) [5]. The NMR spectra for compounds **1** (Figures S1 and S2), **3** (Figures S3 and S4), **7** (Figures S5 and S6) and **9** (Figures S7 and S8) are presented in the Supplementary Materials.

### 2.3. Sargaquinoic Acid (**3**) and Sarganaphthoquinoic Acid (**10**)

To a solution of **1** (154.0 mg, 0.36 mmol) in a mixture of CHCl_3_ (8 mL) and MeOH (7 mL) was added Ag_2_O (100 mg, 0.43 mmol). The reaction mixture was stirred at room temperature for 24 h, after which the resulting suspension was filtered through diatomaceous earth and concentrated under reduced pressure. The crude product was filtered through a plug of charcoal (*n*-hexane-EtOAc, 4:6) to give a yellow mixture of compounds which was separated by silica gel column chromatography (*n*-hexane-EtOAc, 7:3) to give sargaquinoic acid (**3**) (80 mg, 70%) and compound **10** (9.8 mg, 6%) as light yellow oils. NMR spectra for compound **10** (Figures S9–S14) can be found in the Supplementary Materials.

Sarganaphthoquinonoic acid (**10**): IR (film) ν_max_ (cm^−1^): 1600, 1663, 2850, 2924; ^1^H and ^13^C NMR data see Table 1; HRESIMS *m/z* 419.2222 [M-H] (calcd. for C_27_H_35_O_3_, 419.2221)

### 2.4. Sargaquinoic Acid Methyl ester (**5**) 

To a solution of **1** (122.4 mg, 0.29 mmol) dissolved in 2 mL acetone, was added K_2_CO_3_ (207.4 mg, 1.50 mmol) in 5 mL acetone and dimethylsulphate (250 µL, 2.63 mmol). The mixture was heated at 40 °C for 8 h followed by stirring at room temperature for 16 h. The reaction mixture was filtered, concentrated under reduced pressure and separated by silica gel column chromatography (*n*-hexane- EtOAc, 8:2) to give the methyl ester of **1**, which, upon exposure to air was completely oxidized to **5**. NMR spectra for compound **5** (Figures S15–S16) can be found in the Supplementary Materials. 

Yellow oil, ^1^H NMR (400 MHz, CDCl_3_) δ 6.52 (1H, s, H-3), 6.44 (1H, s, H-5), 5.83 (1H, t, *J* = 7.0 Hz, H-10’), 5.10 (3H, m, H-2’, H-6’, H-14’), 3.71 (3H, s, OMe), 3.11 (2H, d, *J* = 6.8 Hz, H-1’), 2.49 (2H, m, H-9’), 2.22 (2H, m, H-12’), 2.05 (2H, m, H-4’) 2.03-2.05 (6H, m, H-5’, H-8’, H-13’), 1.65 (6H, s, H-7, H-19’), 1.61 (3H, s, H-20’), 1.58 (3H, s, H-16’), 1.55 (3H, s, H-17’); 188.0 (C-1, C-4), 168.4 (C-18’), 148.4 (C-6), 145.8 (C-2), 142.1 (C-10’), 140.0 (C-3’) 134.8 (C-7’) 133.1 (C-3, C-15’), 132.1 (C-5), 131.4 (C-11’), 124.4 (C-6’), 123.5 (C-14’), 118.0 (C-2’), 51.0 (OMe), 39.8 (C-4’), 39.1 (C-8’), 34.7 (C-12’), 28.0 (C-9’), 27.8 (C13’), 27.5 (C-1’), 26.4 (C-5’), 25.6 (C-16’), 17.6 (C-17’), 16.1 (C-7), 15.9 (C-19’); HRESIMS *m/z* 437.2710 [M-H] (calcd. for C_28_H_37_O_4_, 437.2692).

### 2.5. Diacetyl Sargahydroquinoic Acid (**2**)

To sargahydroquinoic acid (**1**) (110.0 mg, 0.26 mmol) was added acetic anhydride (3 mL, 31.8 mmol) and pyridine (2 mL, 24.8 mmol). The reaction mixture was stirred at room temperature for 30 h. The crude product was acidified with 1 M HCl (10 mL) and extracted with EtOAc (5 mL × 3). The organic layer was collected and concentrated under reduced pressure to give a crude product which was further purified by silica gel column chromatography (*n*-hexane:EtOAc, 7:3) to give compound **2** (12.8 mg, 12%) as a yellow oil. The structure of compound **2** was confirmed by spectroscopic methods, which were in agreement with literature data [6,7]. NMR spectra for compound **2** (Figures S17 and S18) can be found in the Supplementary Materials.

### 2.6. Sargaquinol (**6**) and Sargachromendiol (**8**)

To a solution of sargahydroquinoic acid (**1**) (140.7 mg, 0.33 mmol) dissolved in anhydrous THF (5 mL), was added LiAlH_4_ (0.104 g, 2.74 mmol). The reaction mixture was stirred at room temperature, under a nitrogen atmosphere for 1.25 h. The reaction was quenched with a few drops of EtOAc, concentrated and partitioned between EtOAc (10 mL^−2^) and H_2_O (5 mL). The organic layer was concentrated under reduced pressure to give a crude product which was purified by silica gel chromatography (*n*-hexane:EtOAc, 8:2) to give sargaquinol (**6**) (12.2 mg, 30%) and the alcohol derivative of sargachromenol (**8**) (2.8 mg, 3.5%). The structure of compound **6** was confirmed by spectroscopic methods, which were in agreement with literature data (Table S1) [7]. NMR spectra for compounds **6** (Figures S19 and S20) and **8** (Figures S21 and S22) can be found in the Supplementary Materials.

Sargaquinol (**6**) yellow oil; ^1^H NMR (400 MHz, CDCl_3_) δ 6.54 (s, 1H) (H-3), 6.46 (s, 1H) (H-5), 5.15 (dd, *J* = 21.0, 13.2 Hz) (H- 2’, 6’, 14’), 4.11 (s) (H-18’), 3.63 (t, *J* = 6.5 Hz) (H-10’), 3.12 (d, *J* = 7.1 Hz) (H-1’), 2.12 (s) (H-5’, 9’, 13’), 2.05 (s) (H-4’, 8’), 1.67 (s) (H- 7, 16’), 1.60 (s) (H-19’, 20’), 1.57 (s) (H-17’). ^13^C NMR (100 MHz, CDCl_3_) δ 188.0 (C-1), 187.97 (C-4), 148.5 (C-6), 145.9 (C-2), 139.7 (C-3’), 135.0 (C-7’), 133.1 (C-3), 131,2 (C-11’), 132.24 (C-5), 133.7 (C-15’), 124.7 (C-6’), 124.2 (C-14’), 118.1 (C-2’), 71.8 (C-18’), 62.8 (C-10’), 39.8 (C-4’), 39.5 (C-8’), 35.2 (C-12’), 27.1 (C-13’),27.5 (C-1’), 26.2 (C-5’), 26.3 (C-9’), 25.6 (C-16’), 17.7 (C-7), 16.11 (C-17’), 16.07 (C-19’), 16.0 (C-20’).

Sargachromendiol (**8**) yellow oil; ^1^H NMR (400 MHz, CDCl_3_) δ 6.47 (d, *J* = 2.4 Hz) (H-5), 6.32 (d, *J* = 2.5 Hz) (H-2), 6.26 (s) (H-2’), 5.98 (t, *J* = 7.2 Hz) (H-9’), 5.57 (d, *J* = 9.8 Hz) (H-3’), 5.12 (dt, *J* = 19.0, 6.4 Hz) (H-5’, 14’), 2.59 (q, *J* = 7.3 Hz (H-8’), 2.27 (t, *J* = 7.4 Hz), 2.13 (s) (H-12’), 2.09–2.04 (m) (H-4’, 7’), 1.68 (s) (H-8), 1.58 (d, *J* = 3.5 Hz) (H-17’, 19’), 1.36 (s) (20’); ^13^C NMR (100 MHz, CDCl_3_) δ 148.6 (C-5), 145.0 (C-10’), 144.8 (C-8), 134.7 (C-7’), 131.8 (C-15’), 130.6 (C-11’), 126.3 (C-3), 124.7 (C-6’), 124.1 (C-1’), 122.9 (C-14’), 121.3 (C-2), 117.0 (C-4), 110.3 (C-6), 77.8 (C-3’), 60.3 (C-18’), 40.7 (C-4’), 39.8 (C-8’), 35.1 (C-12’), 35.3 (C-12’), 27.0 (C-9’), 26.1 (C-13’), 25.9 (C-20’), 25.7 (C-16’), 22.6 (C-5’), 17.7 (17’), 15.9 (C-7), 15.5 (C-19’).

### 2.7. Z-sargaquinal (**4**)

To a solution of sargaquinol (**6**) (37.2 mg, 0.09 mmol) dissolved in anhydrous CH_2_Cl_2_ (8 mL), Dess-Martin Periodinane (107 mg, 0.26 mmol) was added. The reaction mixture was stirred at room temperature for 2 h after which it was quenched with CH_2_Cl_2_ (10 mL) and de-ionized water (10 mL). The organic phase was separated and washed with saturated solutions of NaHCO_3_ (10 mL × 3) and Na_2_S_2_O_3_ (10 mL × 3), dried over anhydrous Na_2_SO_4_ and concentrated under reduced pressure. The crude product was purified by silica gel column chromatography (*n*-hexane:EtOAc, 8:2) to give compound **4** (42%, 14.9 mg), as a yellow oil. The structure of compound **4** was confirmed by spectroscopic methods, which were in agreement with literature data (Table S1) [7]. NMR spectra for compound **4** (Figures S23–S24) can be found in the Supplementary Materials.

### 2.8. Antiplasmodial Assays

All compounds were tested in triplicate against the chloroquine-resistant Gambian FCR-3 strain of *P. falciparum*. The in vitro erythrocytic stage of the parasite was maintained using the method outlined by Trager and Jensen [8]. The antimalarial activity of the compounds was determined using the tritiated hypoxanthine incorporation assay using a 0.5% parasitaemia and 1% haematocrit [9]. All assays were carried out using untreated parasites and uninfected red blood cells as controls. The concentration that inhibited 50% parasite growth (IC_50_ value) was determined from the log sigmoid dose response curve using GraphPad Prism. Quinine was used as the reference antiplasmodial agent. The selectivity index for the compounds was determined from the ratio of cytotoxicity IC_50_ to antimalarial IC_50_.

### 2.9. Cytotoxicity Assay

All compounds were tested in triplicate against MDA-MB-231 breast carcinoma cells, which were purchased from the ATCC (Catalogue number HTB-26, Manassas, VA, USA). The cytotoxicity of the compounds was determined using the WST-1 assay method (Roche). The cells were treated with a range of concentrations of the test compounds or vehicle control (DMSO). Cells treated with DMSO were considered to represent 100% viability and the viability of cells at each dose was represented relative to this value. The concentration resulting in a decrease of cell viability to 50% was calculated from the linear portion of the dose response curve.

## 3. Results and Discussion

### 3.1. Isolation and Synthetic Modification of Sargahydroquinoic Acid Derivatives

Sargahydroquinoic acid (**1**) is the major component of the CH_2_Cl_2_-MeOH extract of *Sargassum incisifolium* and has also been reported from several other *Sargassum* spp. [5,6,7]. This compound slowly converts to sargaquinoic acid (**3**) and sargachromenol (**7**) on storage of the seaweed, the extract and during purification. Specimens of *S. incisifolium* (PA071b) were collected from Port Alfred on the south eastern coast of South Africa and extracted with CH_2_Cl_2_-MeOH. The crude extract was first fractionated by silica gel column chromatography, followed by normal or reversed phase HPLC to give compounds **1**, **3**, **7** and **9**. The identities of all isolated compounds were confirmed by comparison of their NMR spectroscopic data (Table S1) to literature values [5]. The above protocol yielded sufficient quantities of **1** to perform structural modifications and biological assays. 

Sargaquinoic acid (**3**) is normally isolated from fresh seaweed in relatively small quantities; however, it can be produced more efficiently by the oxidation of **1 [7,10]**. Thus, treatment of **1** with Ag_2_O gave **3** in moderate to good yields. Interestingly, although this conversion is facile, we consistently observed a series of unusual peaks between δ_H_ 7 and 8 in the ^1^H NMR spectrum of the crude reaction product. The compound (**10**) responsible for these peaks was isolated and its structure was elucidated by NMR spectroscopy and mass spectrometry. 

**Figure 2:**
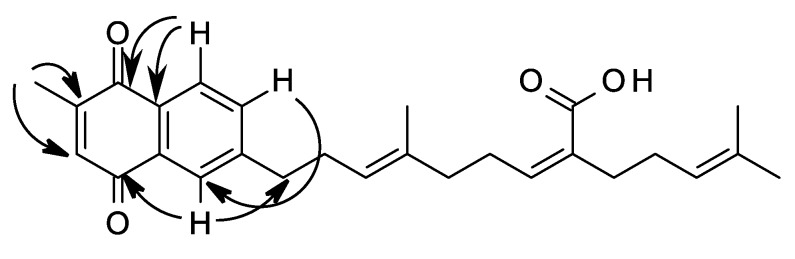
Key HMBC correlations for **10**.

The HRESIMS spectrum of compound **10** showed a molecular ion peak at *m/z* 419.2222 [M-H] which corresponds to a molecular formula of C_27_H_31_O_4_. Characteristic deshielded methine resonances at δ_H_ 8.00 (d, *J* = 7.9), δ 7.98 (s) and δ 7.51 (d, *J* = 7.9) were evident in its ^1^H NMR spectrum. In addition, one of the aromatic singlets had shifted downfield from δ_H_ 6.46 in **3** to δ_H_ 6.81 in **10**. Data from the ^13^C NMR spectrum of compound **10** revealed no change in the number of carbon atoms when compared to the starting material (**1**). It revealed the presence of two quinone carbonyls signals at (δ_C_ 185.4 and δ 185.4) and a carboxylic acid moiety (δ_C_ 171.9). In addition, the DEPT-135 NMR spectrum indicated the loss of one methyl signal (δ_C_ 16.1, C-20’) and a methylene signal (δ_C_ 27.5, C-1’) when compared to **3**, together with the appearance of two additional olefinic methine signals at δ_C_ 126.7 (C-1’) and 125.9 (C-20’). HMBC correlations (Figure 2) from the doublet at δ_H_ 8.00 (H-1’) to carbon signals at δ_C_ 126.7 (C-1’) and δ_C_ 133.8 (C-2’); the methine signal at δ_H_ 7.51 (H-2’) to the carbon signal at δ_C_ 125.9 (C-20’) and from δ_H_ 7.86 (H-20’) to carbon signals at δ_C_ 36.2 (C-4’) and δ_C_ 185.4 (C-4), allowed for the assignment of the naphthoquinone moiety. All other spectroscopic data are consistent with a polyprenyl side chain with a 6’*E*,10’*Z*-double bond geometry (as in **3**). We assigned the name sarganaphthoquinoic acid to this new compound. A related compound, chabrolonaphthoquinone, had previously been reported from the Taiwanese soft coral, *Nephthea chabrolii* [11]. The main differences between the two compounds are the methyl substituent at C-6 and the 10-double bond geometry in compound **10**. 

**Scheme 1:**
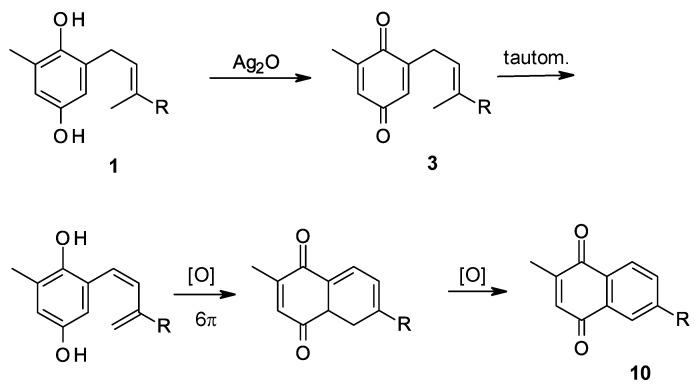
Proposed mechanism for the synthesis of sarganaphthoquinoic acid (**10**).

The direct conversion of prenylated hydroquinones to naphthoquinones is uncommon and presents a novel approach to the synthesis of this important group of compounds. To the best of our knowledge there is only a single report describing the formation of a naphthoquinone as a side-product in the synthesis of chromenes from prenylated quinones [12]. Naphthoquinones are typically synthesized by Diels-Alder reactions between *p*-benzoquinones and dienes or by the prenylation of halogenated naphthoquinone moieties [13,14,15]. Compound **10** is proposed to form via tautomerism and oxidation of the intermediate quinone (**3**) followed by 6πelectrocyclization and further oxidation (Scheme 1). 

In order to establish preliminary structure-antiplasmodial activity relationships for this series of sargahydroquinoic acid derivatives, we focused our attention on modification of the carboxylic acid and quinone moieties. Acetylation of **1** with acetic anhydride/pyridine gave the diacetate (**2**), while its reduction with lithium aluminium hydride gave a mixture of sargaquinol (**6**) and sargachromendiol (**8**). The facile conversion of the hydroquinone to a mixture of the quinone and chromene on exposure to air is often seen in this series of compounds [6,7]. Spectroscopic evidence for the identity of alcohols **6** and **8** were provided by the disappearance of the ^13^C NMR signal due to the carboxylic acid group at δ_C_ 172 ppm and the appearance of an oxymethylene carbon signal at δ_C_ 60.3 ppm in both compounds. 

Mild oxidation of **6** with Dess-Martin periodinane, gave 10*Z*-sargaquinal (**4**). The structures of aldehydes **4** and **9** were confirmed by comparison of their spectroscopic data with literature values [5,7]. A comparison of the ^1^H NMR spectra of the natural and semi-synthetic aldehydes revealed differences in chemical shifts of both proton and carbon atoms associated with the aldehyde group. The ^1^H and ^13^C NMR spectra of the semi-synthetic aldehyde (**4**) showed signals at δ_H_ 10.1 and δ_C_ 190.9 ppm compared to δ_H_ 9.55 and δ_C_ 205.4 ppm in the natural aldehyde (**9**). ^1^H-^1^H NOESY correlations in both compounds confirmed the difference in the geometry of the Δ^10^ double bond with the semi-synthetic aldehyde (**4**) bearing a 10*Z*-geometry and the natural aldehyde (**9**) a 10*E*-geometry. The formation of 2’*E*,6’*E*,10’*Z*-sargaquinal (**4**) from 2’*E*,6’*E*,10’*Z*-sargahydroquinoic acid (**1**) has been reported in the literature [7]. However, this is the first report of its ^13^C and 2D NMR data.

Interestingly, methylation of **1** with dimethylsulphate/potassium carbonate did not produce the dimethyl ether, but instead produced sargaquinoic acid methyl ester (**5**). This was confirmed by the appearance of an additional methyl signal at δ_C_ 51.0 and an upfield shift of the C-18’ carbonyl signal from δ_C_ 172 to δ 168.4 ppm in the ^13^C NMR spectrum of **5** (Table S1). 

### 3.2. Biological Assays

The ten sargahydroquinoic acid derivatives were assessed for both antiplasmodial and cytotoxic activity against the chloroquine-resistant Gambian FCR-3 strain of *P. falciparum* and MDA-MB-231 breast cells, respectively (Table 2). All compounds showed moderate to good antiplasmodial activity. However, the most promising compound in this series is the naphthoquinone **10** which not only revealed good antiplasmodial activity (IC_50_ 5.4 μM), but also very low cytotoxicity (IC_50_ 2410 μM), resulting in a high selectivity index of 443. Sargaquinoic acid (**3**) also shows promising antiplasmodial activity (IC_50_ 10.8 μM), but is slightly more toxic (IC_50_ 658 μM) than **10**. It appears that the carboxylic acid in the prenyl side chain is important for activity since both aldehydes (**4**) and (**9**) and the alcohol (**6**) showed decreased antiplasmodial activity. The quinone/naphthoquinone scaffold is present in several antimalarial natural products and drugs [16]. It is therefore likely that the mode of action of the compounds reported here is related to this important pharmacophore [16,17,18,19,20]. 

## 4. Conclusions

In this study we isolated the relatively abundant antiplasmodial natural product, sargahydroquinoic acid (**1**) and converted it to several analogs which were evaluated for antiplasmodial and cytotoxic activity. The serendipitous formation of sarganaphthoquinoic acid (**10**) gave a compound with good antiplasmodial activity while being almost non-toxic. Due to the small number of compounds no clear structure activity relationships can be established, however it appears that the presence of a quinone and carboxylic acid are important for selective activity against *P. falciparum*. Further studies are warranted to explore the mode of action of these compounds and to further improve on its antiplasmodial activity.

## Figures and Tables

**Table 1:** NMR spectroscopic data for sarganaphthoquinoic aicd (**10**) (600 and 125 MHz, CDCl_3_).

Carbon Number	δ_C_	Type	δ_H_, mult, *J* (Hz)	COSY	HMBC
1	185.4	C	-		
2	130.2	C	-		
3	132.2	C	-		
4	185.4	C	-		
5	136.0	CH	6.81, s	H-7	
6	149.0	C	-		
7	16.4	CH_3_	2.18, s,	H-5	C-6, C-5
1’	126.7	CH	8.00, d, 7.9	H-2’	C-2, C-1
2’	133.8	CH	7.51, d, 7.9	H-4’, H-20’	C-1, C-20’
3’	148.2	C	-		
4’	36.2	CH_2_	2.77, t, 7.6	H-5’	C-5’, C-3’
5’	29.1	CH_2_	2.36, m	H-4’, H-6’	C-4’, C-6’, C-7’
6’	123.2	CH	5.17, m		
7’	136.0	C	-		
8’	39.0	CH_2_	2.08, m	H-9’	C-6’
9’	28.2	CH_2_	2.57, m	H-10’	C-8’, C-10’
10’	145.0	CH	5.96, t, 7.3	H-9’	C-8’
11’	130.6	C	-		
12’	27.8	CH_2_	2.26, m	H-13’, H-14’ (lr)	C-13’, C-14’
13’	28.2	CH_2_	2.11, m	H-14’	C-15’, C-11’
14’	123.4	CH	5.17, t, 7.0	H-13’, H-14’ (lr)	
15’	132.3	C	-		
16’	25.6	CH_3_	1.68, s	H-14’ (lr)	C-15’, C-14’
17’	17.7	CH_3_	1.59, s	H-14’ (lr)	C-16’
18’	171.9	C	-		
19’	15.9	CH_3_	1.58, s	H-6’	C-6’
20’	125.9	CH	7.86, s	H-4’	C-4’, C-4

COSY: ^1^H-^1^H Correlation spectroscopy; HMBC: ^1^H-^13^C Heteronuclear multiple-bond correlation spectroscopy.

**Table 2:** Bioassay results for compounds **1**–**10**.

Compound	IC_50_ (μM)			Selectivity Index
	D10 ^1^	FCR-3	MDA-MB-231	
Sargahydroquinoic acid (**1**)	15.2	38.6	70	1.8
Sargahydroquinoic acid di-acetate (**2**)	-	84.3	286	3.4
Sargaquinoic acid (**3**)	12.0	10.8	658	60.9
10*Z*-sargaquinal (**4**)	-	72.6	211	2.9
Sargaquinoic acid methyl ester (**5**)	-	8.2	70	8.6
Sargaquinol (**6**)	-	93.1	99	1.1
Sargachromenol (**7**)	-	114.8	56	0.5
Sargachromendiol (**8**)	-	34.2	187	5.5
10*E*-sargaquinal (**9**)	2.0	104.4	69	0.7
Sarganaphthoquinone (**10**)	-	5.4	2410	443
Quinine		0.17	-	-

^1^ From reference [5].

## Supplementary Materials

The following are available online at https://www.mdpi.com/2305-6320/6/2/47/s1, Scheme S1: Isolation of compounds **1**, **3**, **7** and **9**, Table S1: Comparison of ^13^C NMR data for compounds **1**, **3**–**9**, Figure S1: ^1^H NMR spectrum of sargahydroquinoic acid (**1**) (400 MHz, CDCl_3_), Figure S2: ^13^C NMR spectrum of sargahydroquinoic acid (**1**) (100 MHz, CDCl_3_), Figure S3: ^1^H NMR spectrum of sargaquinoic acid (**3**) (400 MHz, CDCl_3_), Figure S4: ^13^C NMR spectrum of compound **3** (400 MHz, CDCl_3_), Figure S5: ^1^H NMR spectrum of sargachromenol (**7**) (400 MHz, CDCl_3_), Figure S6: ^13^C NMR spectrum of sargachromenol (**7**) (100 MHz, CDCl_3_), Figure S7: ^1^H NMR spectrum of 10’E-sargaquinal (**9**) (400 MHz, CDCl_3_), Figure S8: ^13^C NMR spectrum of 10’E-sargaquinal (**9**) (100 MHz, CDCl_3_), Figure S9: ^1^H NMR spectrum of sarganaphthoquinoic acid (**10**) (400 MHz, CDCl_3_), Figure S10: ^13^C NMR spectrum of sarganaphthoquinoic acid (**10**) (100 MHz, CDCl_3_), Figure S11: DEPT-135 NMR spectrum of sarganaphthoquinoic acid (**10**) (100 MHz, CDCl_3_), Figure S12: HSQC NMR spectrum of sarganaphthoquinoic acid (**10**) (CDCl_3_), Figure S13: COSY NMR spectrum of sarganaphthoquinoic acid (**10**) (CDCl_3_), Figure S14: HMBC NMR spectrum of sarganaphthoquinoic acid (**10**) (CDCl_3_). Figure S15: ^1^H NMR spectrum of sargaquinoic acid methyl ester (**5**) (400 MHz, CDCl_3_), Figure S16: ^13^C NMR spectrum of sargaquinoic acid methyl ester (**5**) (100 MHz), Figure S17: ^1^H NMR spectrum of sargahydroquinoic acid diacetate (**2**) (400 MHz, CDCl_3_), Figure S18: ^13^C NMR spectrum of sargahydroquinoic acid diacetate (**2**) (100 MHz, CDCl_3_), Figure S19: ^1^H NMR spectrum of sargaquinol (**6**) (400 MHz, CDCl_3_), Figure S20: ^13^C NMR spectrum of sargaquinol (**6**) (100 MHz, CDCl_3_), Figure S21: ^1^H NMR spectrum of sargachromendiol (**8**) (400 MHz, CDCl_3_), Figure S22: ^13^C NMR spectrum of sargachromendiol (**8**) (100 MHz, CDCl_3_), Figure S23: ^1^H NMR spectrum of 10’Z-sargaquinal (**4**) (600 MHz, CDCl_3_), Figure S24: ^13^C NMR spectrum of 10’Z-sargaquinal (**4**) (100 MHz, CDCl_3_).
