# Supplementary material for: Semi-Synthesis and Evaluation of Sargahydroquinoic Acid Derivatives as Potential Antimalarial Agents
Source: Medicines (Basel). 2019 Apr 1;6(2):47. doi: 10.3390/medicines6020047 (PMC6630221; doi:10.3390/medicines6020047)
Supplement: Supplementary file 1 [file medicines-06-00047-s001.pdf]

Semi-synthesis and evaluation of sargahydroquinoic acid derivatives as potential antimalarial agents

Tatenda C. Munedzimwe [1], Robyn L. van Zyl [2], Donovan C. Heslop [2], Adrienne L. Edkins [3] and Denzil R. Beukes [4*]

[1] Faculty of Pharmacy, Rhodes University, Grahamstown, 6139, South Africa; tatendamunedzimwe@gmail.com

[2] Pharmacology Division, Department of Pharmacy and Pharmacology; WITS Research Institute for Malaria (WRIM); MRC Collaborating Centre for Multidisciplinary Research on Malaria, Faculty of Health Sciences, University of the Witwatersrand, Johannesburg, South Africa; robyn.vanzyl@wits.ac.za, donoheslop@gmail.com

[3] Biomedical Biotechnology Research Unit (BioBRU), Department of Biochemistry and Microbiology, Rhodes University, Grahamstown, 6139, South Africa; a.edkins@ru.ac.za

[4] School of Pharmacy, University of the Western Cape, Bellville, 7535, South Africa; dbeukes@uwc.ac.za

Supplementary data

Contents

Scheme S1. Isolation of compounds **1**, **3**, **7** and **9**......3

Table S1. Comparison of ^{13}C NMR data for compounds **1**, **3-9**......4

Figure S1. ^{1}H NMR spectrum of sargahydroquinoic acid (**1**) (400 MHz, $CDCl_3$)......5

Figure S2. ^{13}C NMR spectrum of sargahydroquinoic acid (**1**) (100 MHz, $CDCl_3$)......5

Figure S3. ^{1}H NMR spectrum of sargaquinoic acid **(3)** (400 MHz, $CDCl_3$)......6

Figure S4. ^{13}C NMR spectrum of compound **3** (400 MHz, $CDCl_3$)......6

Figure S5. ^{1}H NMR spectrum of sargachromenol (**7**) (400 MHz, $CDCl_3$)......7

Figure S6. ^{13}C NMR spectrum of sargachromenol (**7**) (100 MHz, $CDCl_3$)......7

Figure S7. ^{1}H NMR spectrum of 10'*E*-sargaquinal (**9**) (400 MHz, $CDCl_3$)......8

Figure S8. ^{13}C NMR spectrum of 10'*E*-sargaquinal (**9**) (100 MHz, $CDCl_3$)......8

Figure S9. ^{1}H NMR spectrum of sarganaphthoquinoic acid (**10**) (400 MHz, $CDCl_3$)......9

Figure S10. ^{13}C NMR spectrum of sarganaphthoquinoic acid (**10**) (100 MHz, $CDCl_3$)......9

Figure S11. DEPT-135 NMR spectrum of sarganaphthoquinoic acid (**10**) (100 MHz, $CDCl_3$)......10

Figure S12. COSY NMR spectrum of sarganaphthoquinoic acid (**10**) ($CDCl_3$)......10

Figure S13. HSQC NMR spectrum of sarganaphthoquinoic acid (**10**) ($CDCl_3$)......11

Figure S14. HMBC NMR spectrum of sarganaphthoquinoic acid (**10**) ($CDCl_3$)......11

Figure S15. ^{1}H NMR spectrum of sargaquinoic acid methyl ester (**5**) (400 MHz, $CDCl_3$)....12

Figure S16. ^{13}C NMR spectrum of sargaquinoic acid methyl ester (**5**) (100 MHz)......12

Figure S17. ^{1}H NMR spectrum of sargahydroquinoic acid diacetate (**2**) (400 MHz, $CDCl_3$) 13
Figure S18. ^{13}C NMR spectrum of sargahydroquinoic acid diacetate (**2**) (100 MHz, $CDCl_3$) 13
Figure S19. ^{1}H NMR spectrum of sargaquinol (**6**) (400 MHz, $CDCl_3$).................................14
Figure S20. ^{13}C NMR spectrum of sargaquinol (**6**) (100 MHz, $CDCl_3$)................................14
Figure S21. ^{1}H NMR spectrum of sargachromendiol (**8**) (400 MHz, $CDCl_3$)........................15
Figure S22. ^{13}C NMR spectrum of sargachromendiol (**8**) (100 MHz, $CDCl_3$).......................15
Figure S23. ^{1}H NMR spectrum of 10'*Z*-sargaquinal (**4**) (600 MHz, $CDCl_3$) 16
Figure S24. ^{13}C NMR spectrum of 10'*Z*-sargaquinal (**4**) (100 MHz, $CDCl_3$) 16

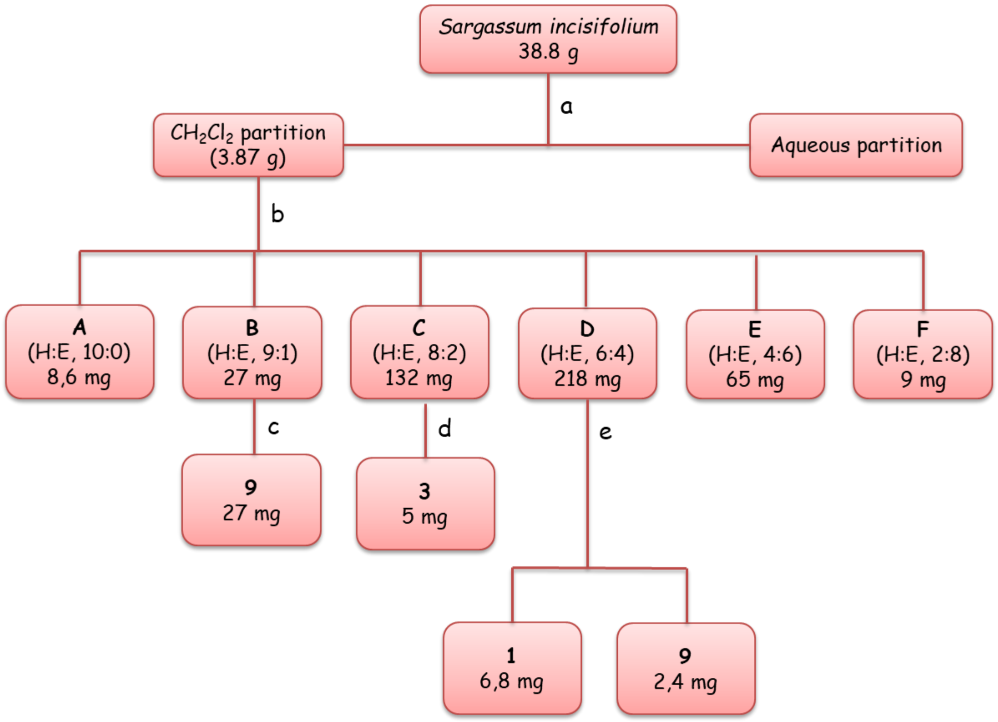

Conditions: a) *i) MeOH extraction, ii) MeOH-CH_2Cl_2 (1:2) extraction, iii) combined extracts, collect CH_2Cl_2, concentrate*, b) *1,09 g extract, silica gel column chromatography (hexane-EtOAc gradient)*, c) *silica gel column chromatography (hexane-EtOAc, 9:1)*, d) *NP (Si) HPLC (hexane-EtOAc, 8:2)*, e) *RP (C18) HPLC (MeOH-H2O, 9:1).*

Scheme S1. Isolation of compounds **1**, **3**, **7** and **9**

Table S1. Comparison of ^{13}C NMR data for compounds **1**, **3-9**

C #	δ_C							
	**1**	**3**	**4**	**5**	**6**	**7**	**8**	**9**
1	146.4	188.0	187.96	187.92	188.0	145.6	-	187.96
2	125.5	145.9	145.9	145.81	145.9	121.3	121.3	145.9
3	115.4	133.1	132.27	133.1	133.1	110.3	126.3	132.3
4	148.7	187.9	187.91	187.87	188.0	148.5	117.0	187.91
5	113.9	132.2	132.12	132.11	132.2	117.1	148.6	132.1
6	127.6	148.5	148.4	148.38	148.5	126.3	110.3	148.4
7	16.1	17.7	15.99	16.07	17.7	15.7	15.9	16.0
1′	29.9	27.5	27.33	27.48	27.5	122.9	124.1	27.3
2′	121.7	117.9	118.2	117.97	118.1	130.7	-	118.2
3′	138.2	139.8	139.9	139.78	139.7	77.8	77.8	139.9
4′	39.5	39.6	39.5	39.56	39.8	40.8	40.7	39.5
5′	26.0	26.3	26.4	26.37	26.2	22.6	22.6	26.4
6′	124.2	124.5	125.5	124.39	124.7	124.9	124.7	125.5
7′	134.7	134.6	133.6	134.6	135.0	134.3	134.8	133.6
8′	39.0	39	39.38	39.09	39.5	39.1	39.8	39.4
9′	28.3	28.2	27.54	27.95	26.3	28.1	27.0	27.5
10′	145.5	145.4	145.9	142.06	-	144.9	145.0	154.9
11′	130.5	130.6	132.1	131.36	131.2	130.5	130.6	132.1
12′	34.5	34.5	27.33	34.66	35.2	34.5	35.1	27.3
13′	27.8	27.9	27.45	27.82	27.1	27.9	26.1	25.7
14′	123.4	123.4	123.6	123.46	124.2	123.4	122.9	123.6
15′	132.3	132.2	133.2	133.1	133.7	132.3	131.8	133.2
16′	25.7	25.6	25.15	25.61	25.6	25.7	25.7	25.2
17′	17.7	16.1	17.72	17.59	16.1	17.7	17.7	17.7
18′	172.4	172.7	190.9	168.42	62.8	172.9	60.3	205.4
19′	16.0	16	16.04	15.93	16.1	15.5	15.5	16.0
20′	16.1	16.1	16.1	15.84	16.0	25.9	25.9	16.1
Me				51.03				

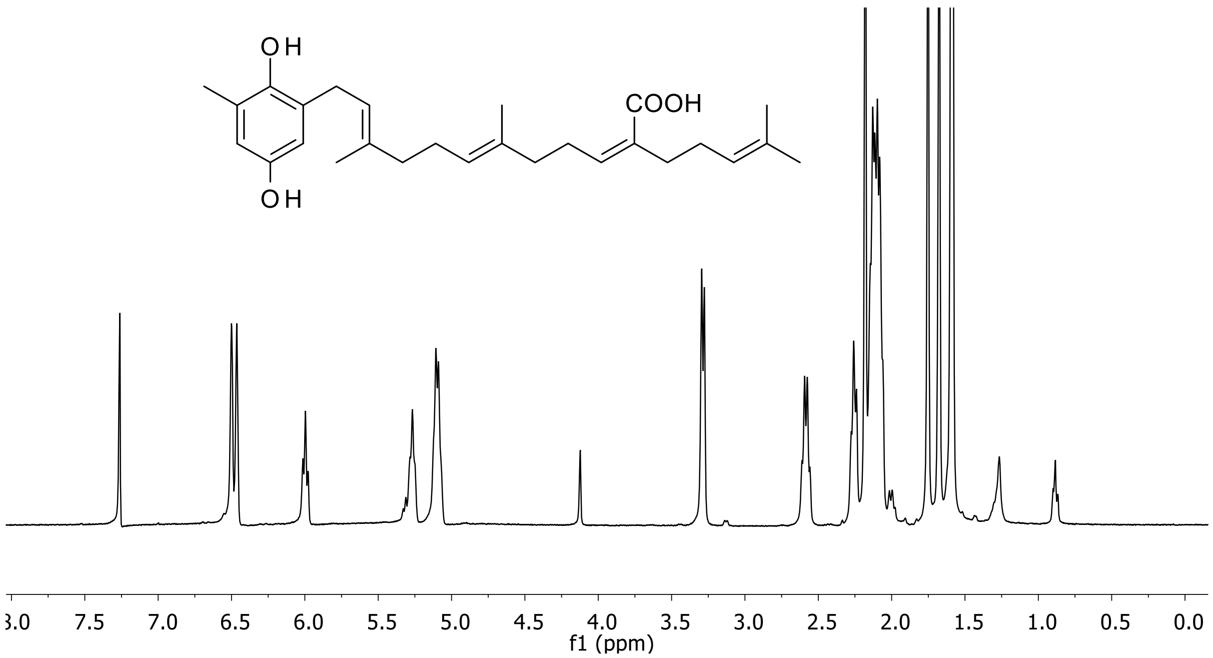

Figure S1. ^{1}H NMR spectrum of sargahydroquinoic acid (**1**) (400 MHz, $CDCl_3$)

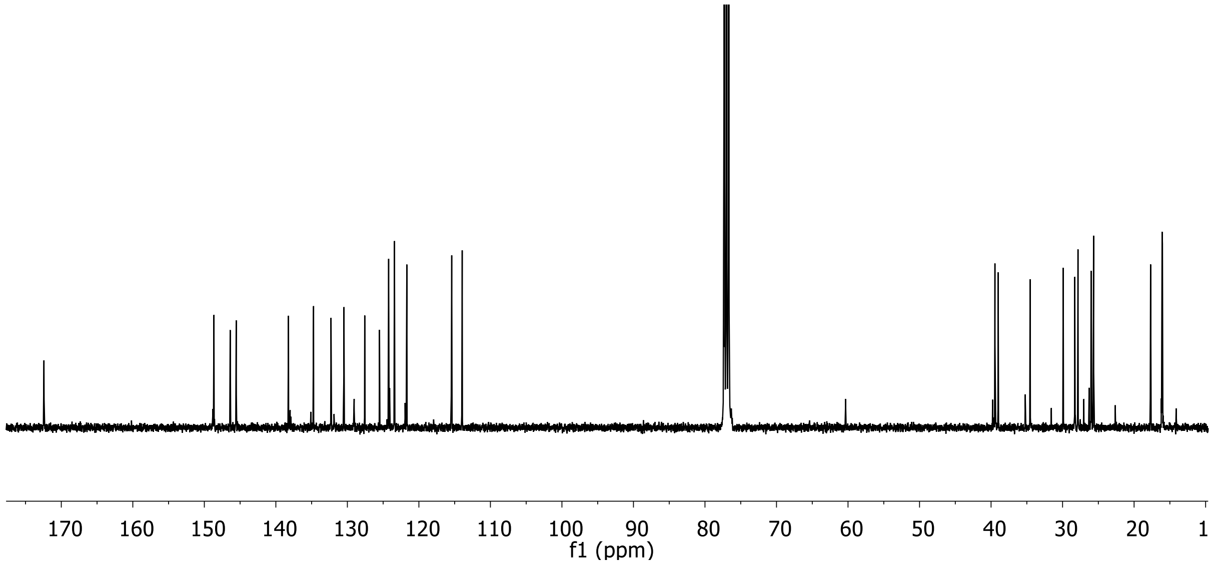

Figure S2. ^{13}C NMR spectrum of sargahydroquinoic acid (**1**) (100 MHz, $CDCl_3$)

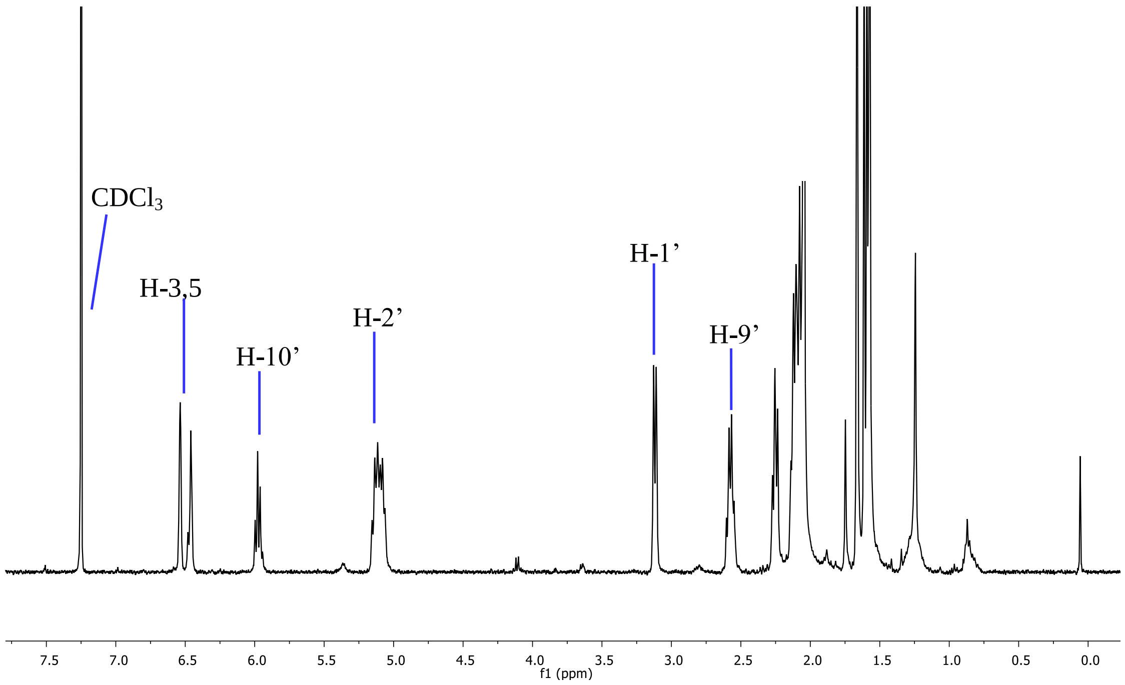

Figure S3. ^{1}H NMR spectrum of sargaquinoic acid **(3)** (400 MHz, $CDCl_3$)

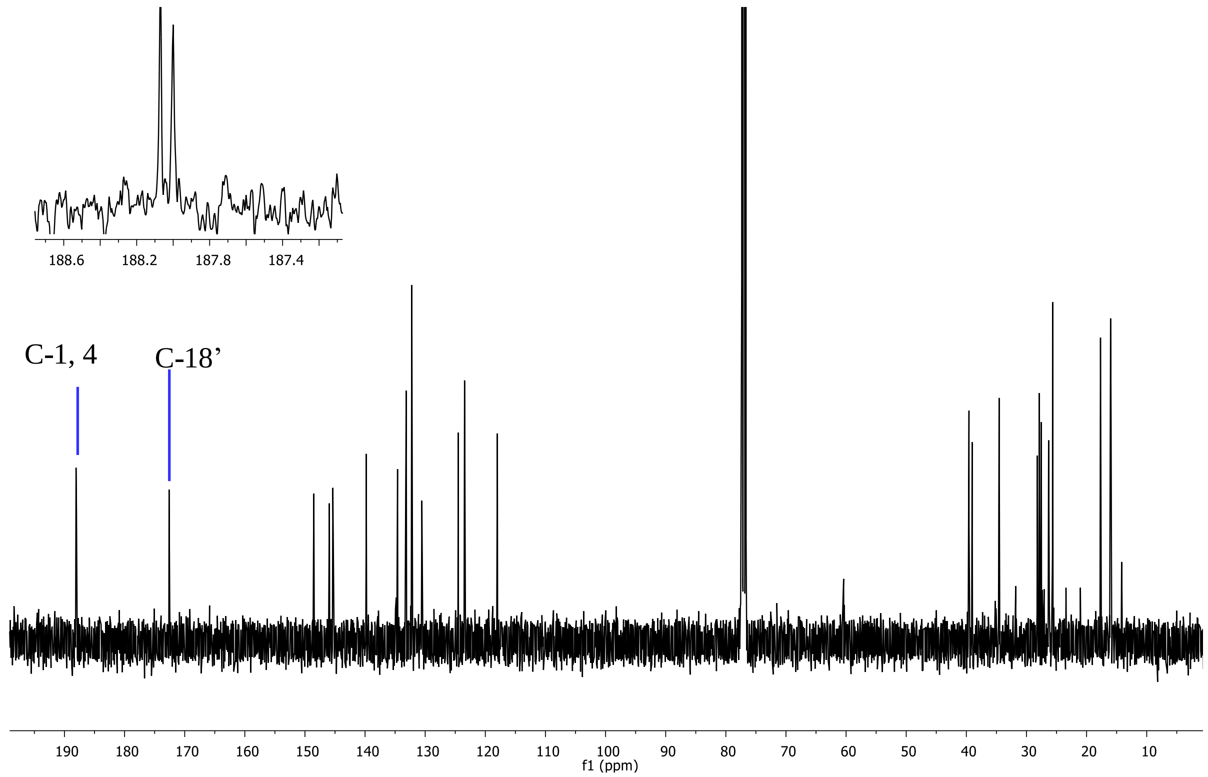

Figure S4. ^{13}C NMR spectrum of compound **3** (400 MHz, $CDCl_3$)

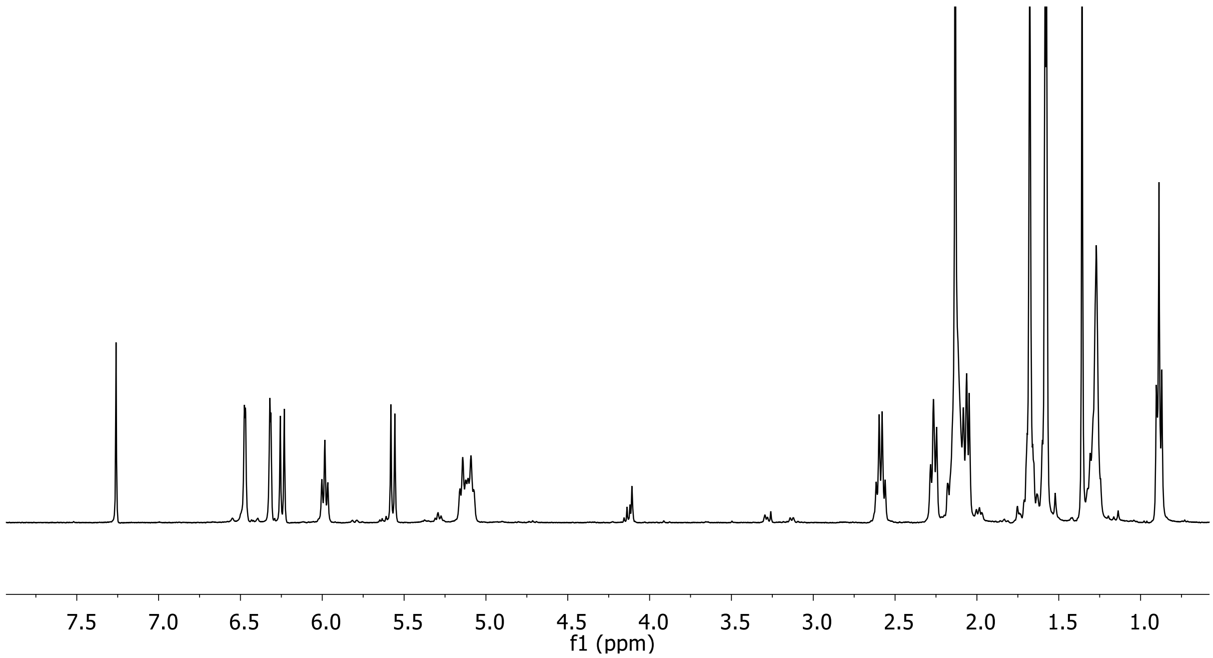

Figure S5. ^{1}H NMR spectrum of sargachromenol (**7**) (400 MHz, $CDCl_3$)

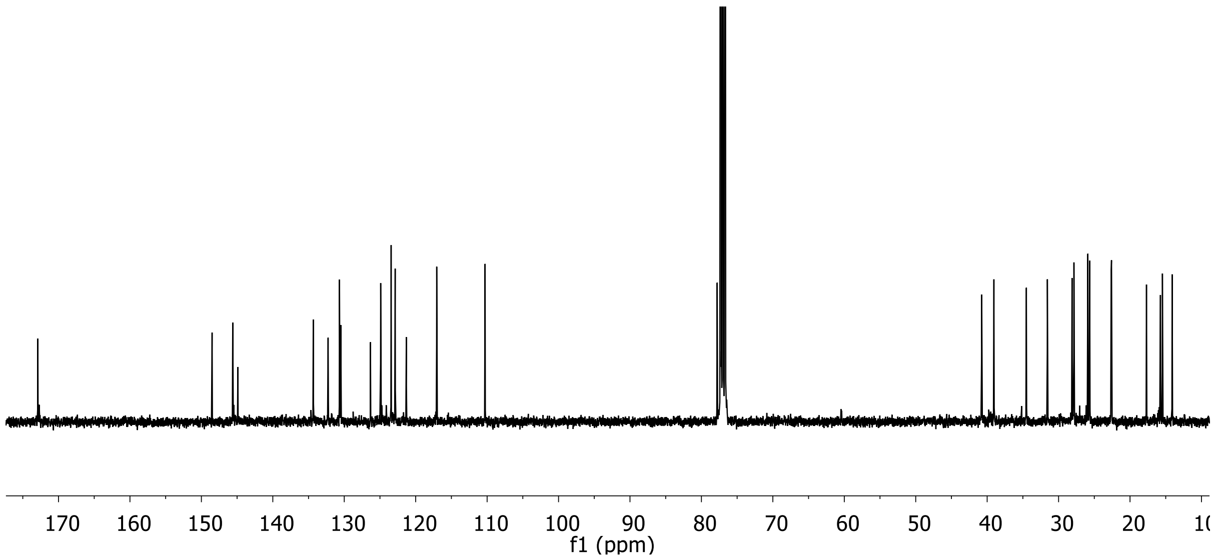

Figure S6. ^{13}C NMR spectrum of sargachromenol (**7**) (100 MHz, $CDCl_3$)

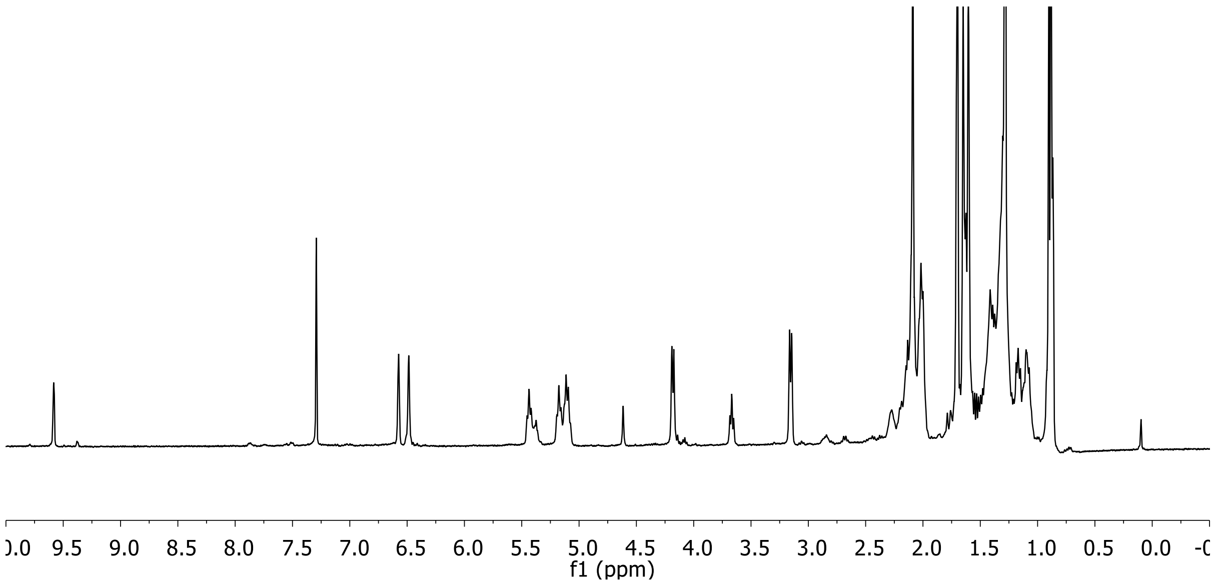

Figure S7. ^{1}H NMR spectrum of 10′*E*-sargaquinal (**9**) (400 MHz, $CDCl_3$)

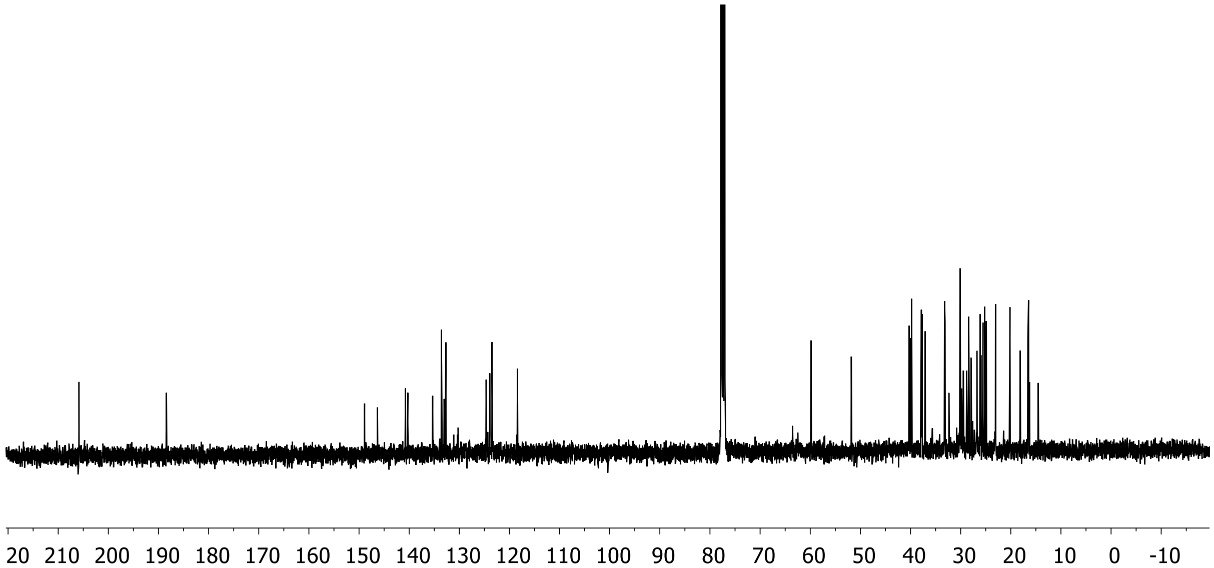

Figure S8. ^{13}C NMR spectrum of 10′*E*-sargaquinal (**9**) (100 MHz, $CDCl_3$)

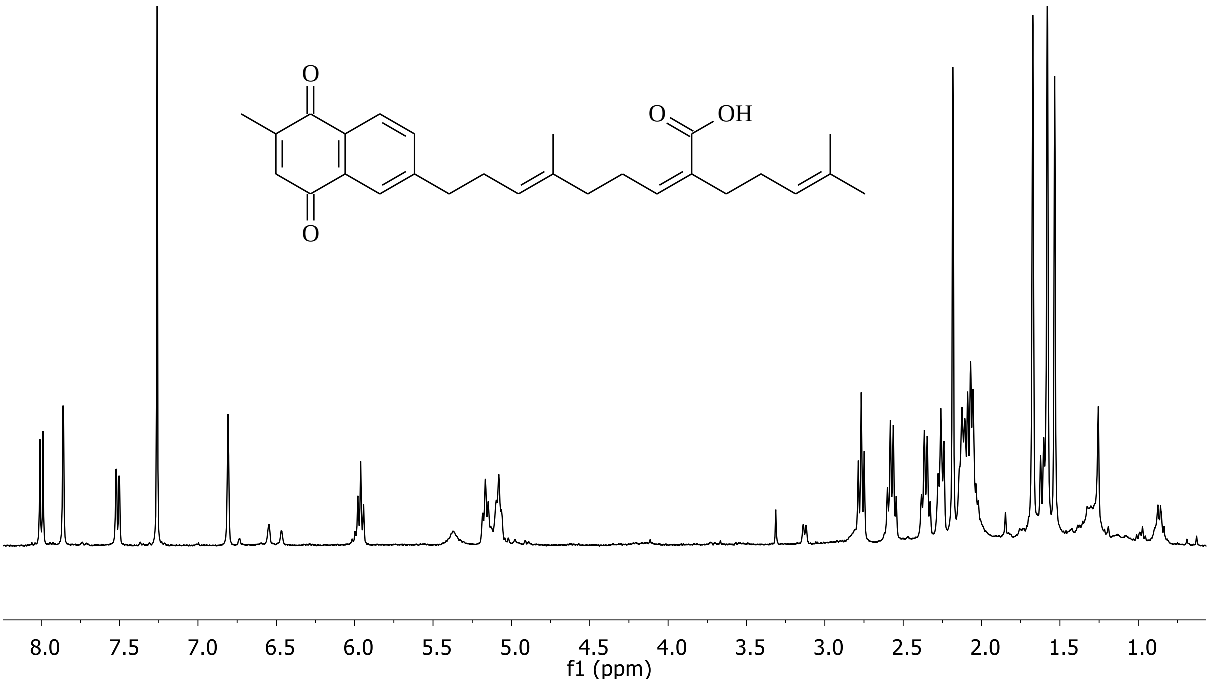

Figure S9. ^{1}H NMR spectrum of sarganaphthoquinoic acid (**10**) (400 MHz, $CDCl_3$)

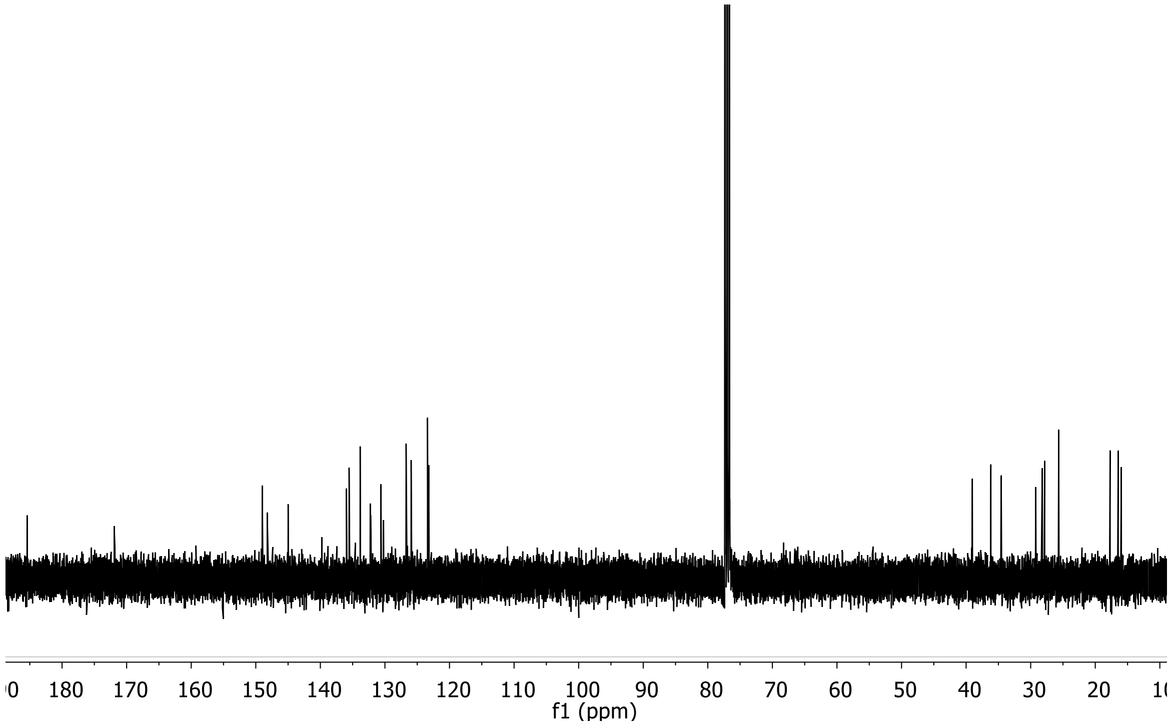

Figure S10. ^{13}C NMR spectrum of sarganaphthoquinoic acid (**10**) (100 MHz, $CDCl_3$)

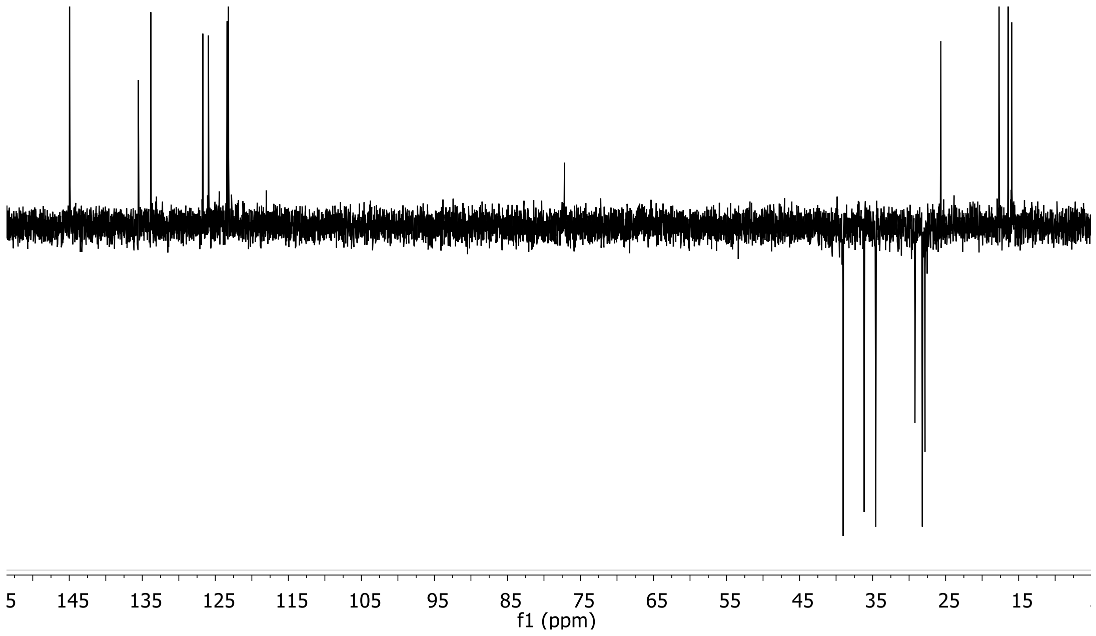

Figure S11. DEPT-135 NMR spectrum of sarganaphthoquinoic acid (**10**) (100 MHz, $CDCl_3$)

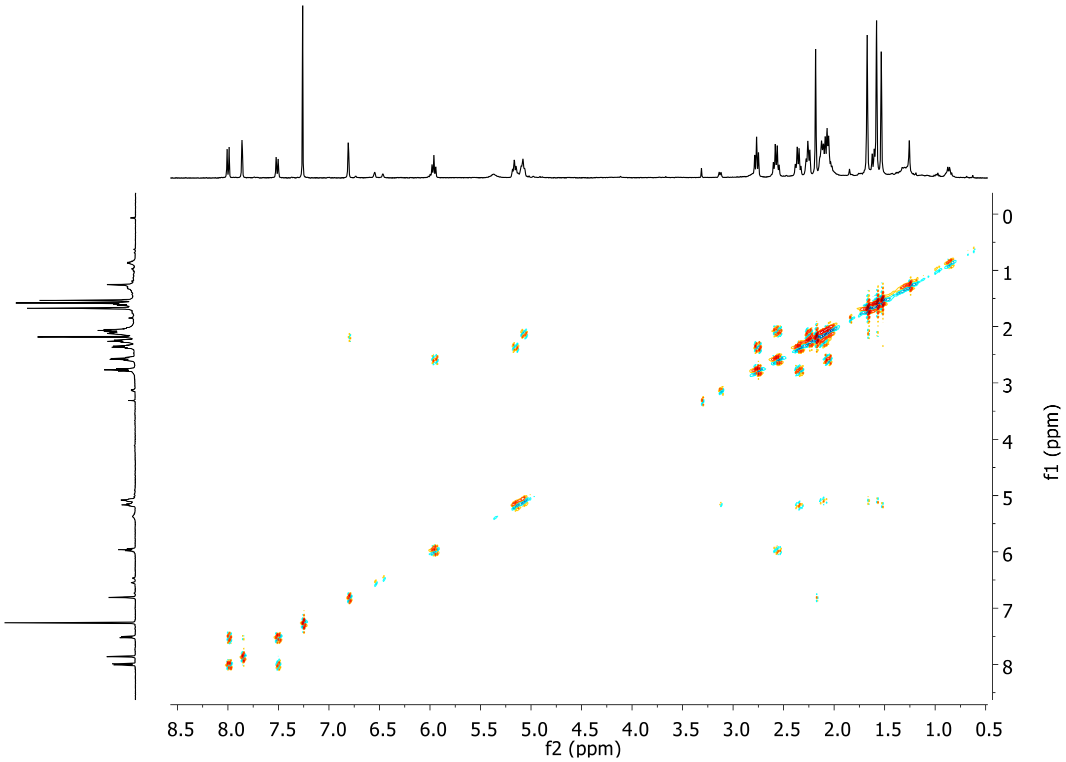

Figure S12. COSY NMR spectrum of sarganaphthoquinoic acid (**10**) ($CDCl_3$)

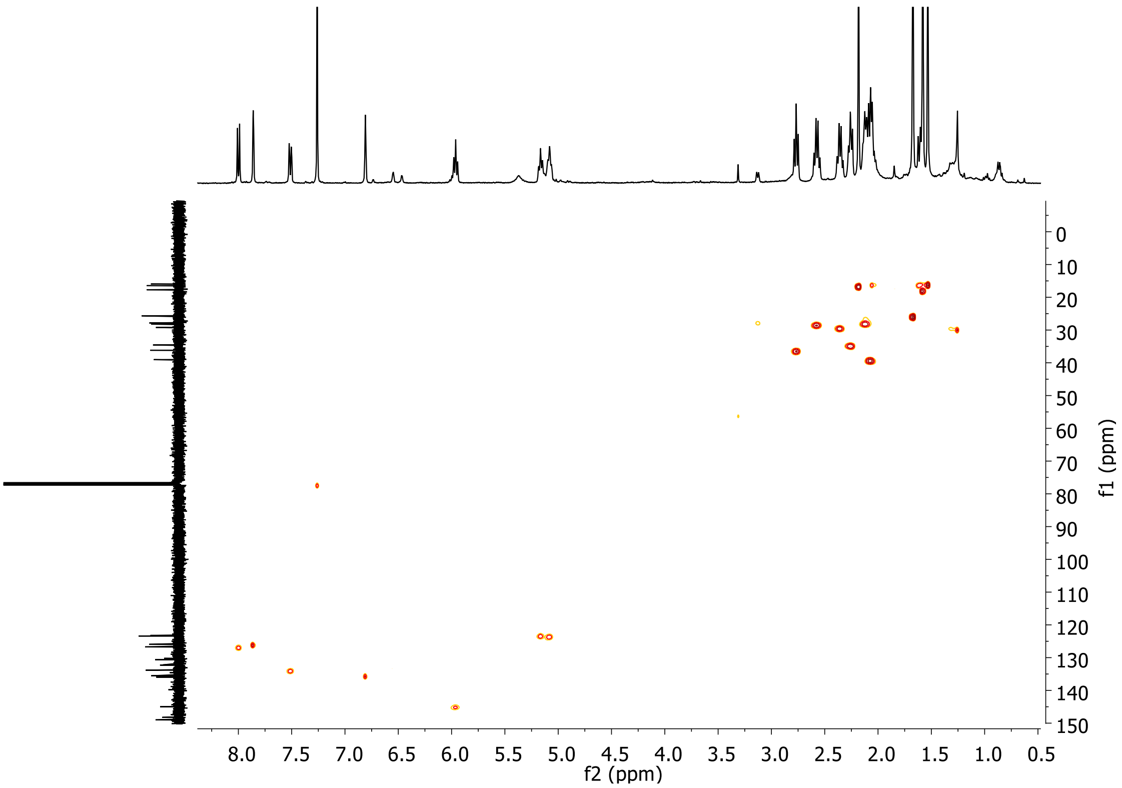

Figure S13. HSQC NMR spectrum of sarganaphthoquinoic acid (**10**) ($CDCl_3$)

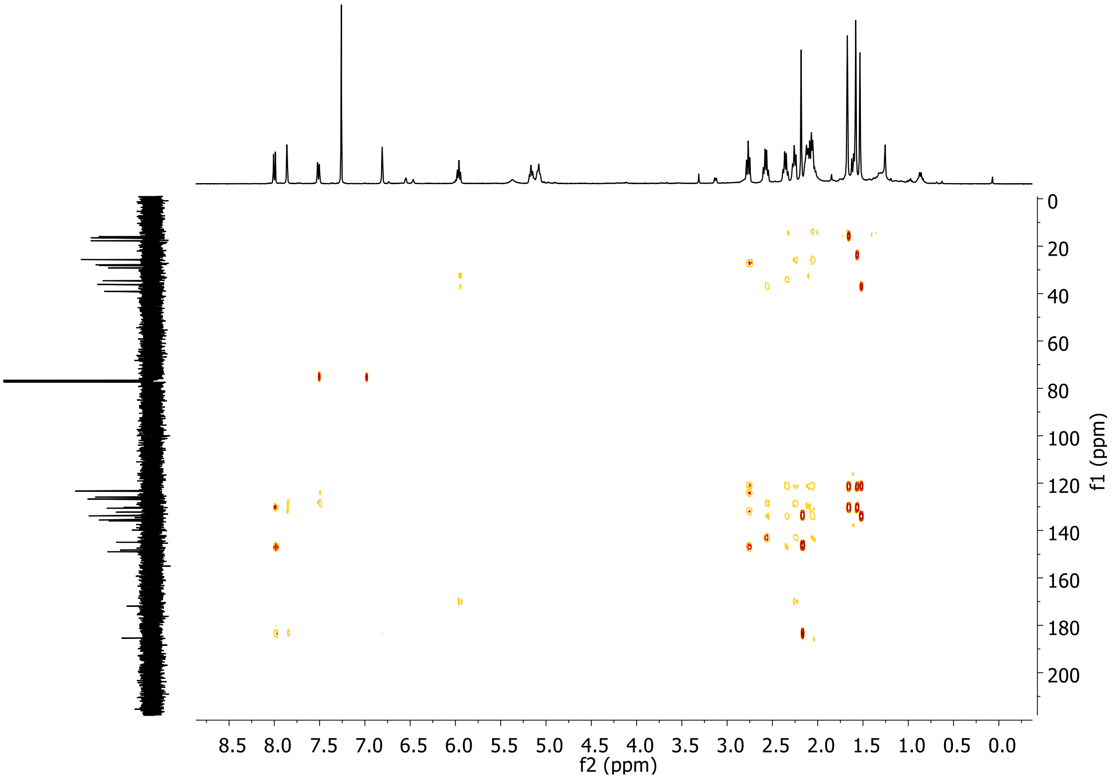

Figure S14. HMBC NMR spectrum of sarganaphthoquinoic acid (**10**) ($CDCl_3$)

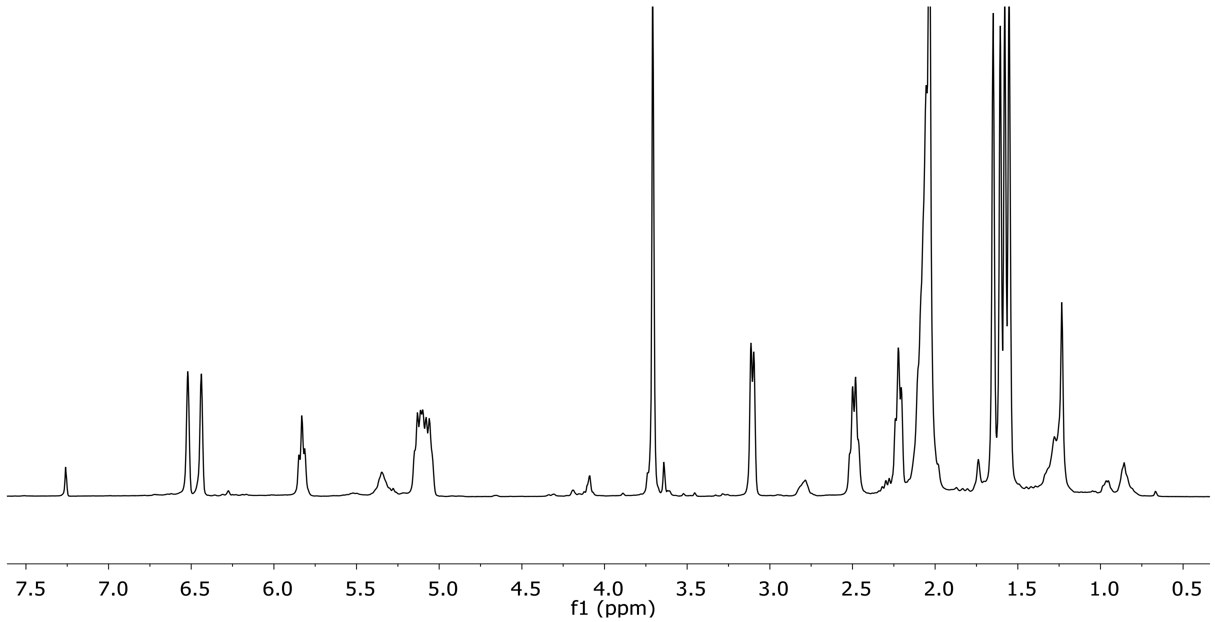

Figure S15. ^{1}H NMR spectrum of sargaquinoic acid methyl ester (**5**) (400 MHz, $CDCl_3$)

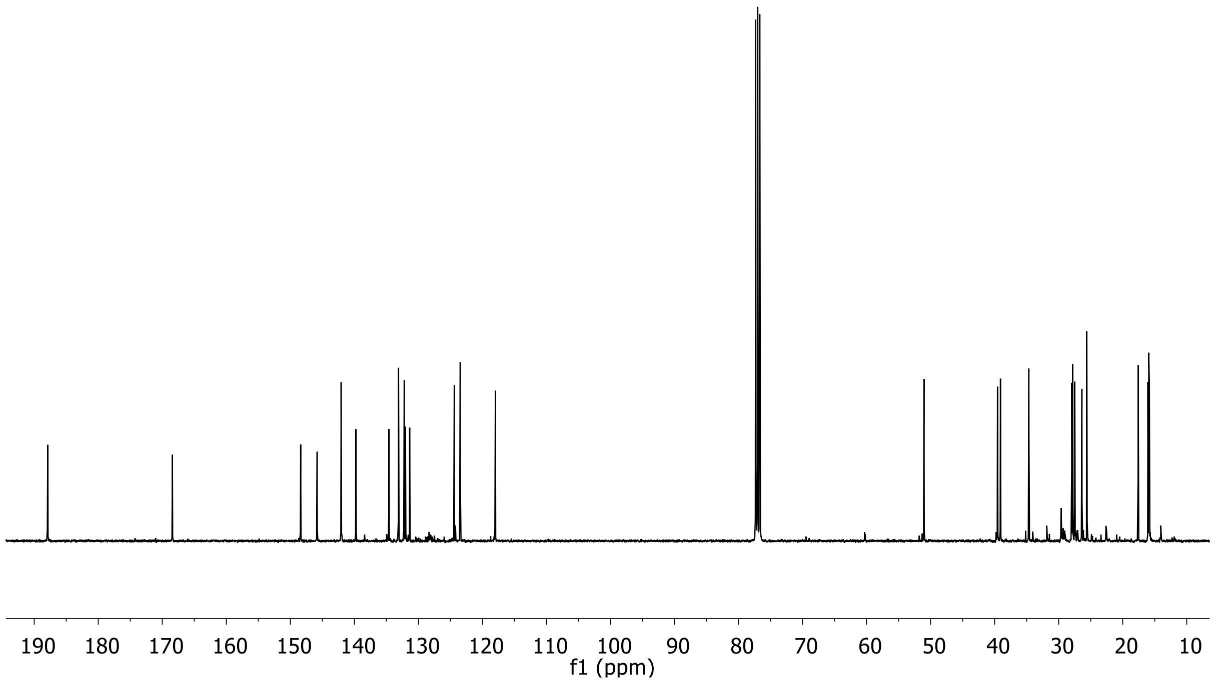

Figure S16. ^{13}C NMR spectrum of sargaquinoic acid methyl ester (**5**) (100 MHz)

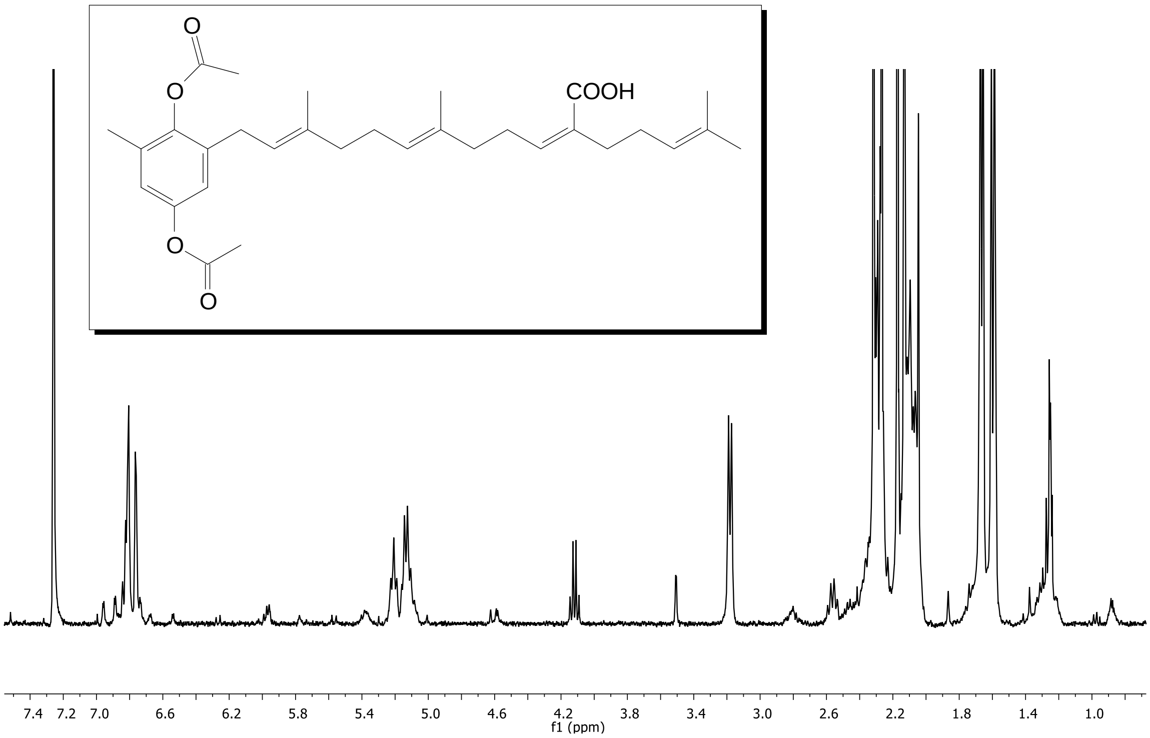

Figure S17. ^{1}H NMR spectrum of sargahydroquinoic acid diacetate (**2**) (400 MHz, $CDCl_3$)

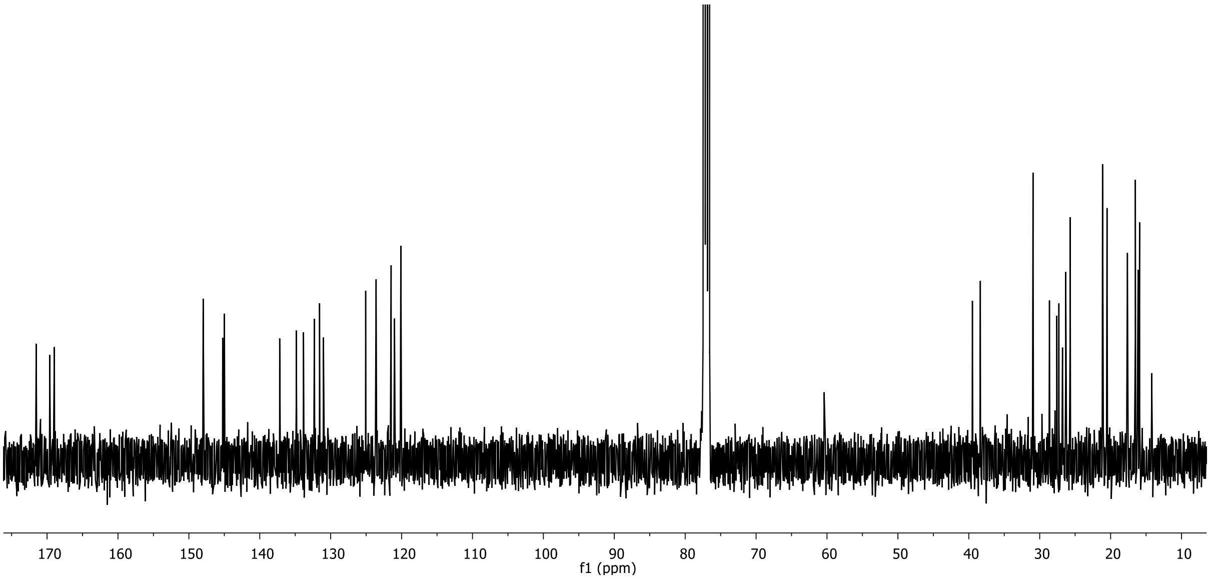

Figure S18. ^{13}C NMR spectrum of sargahydroquinoic acid diacetate (**2**) (100 MHz, $CDCl_3$)

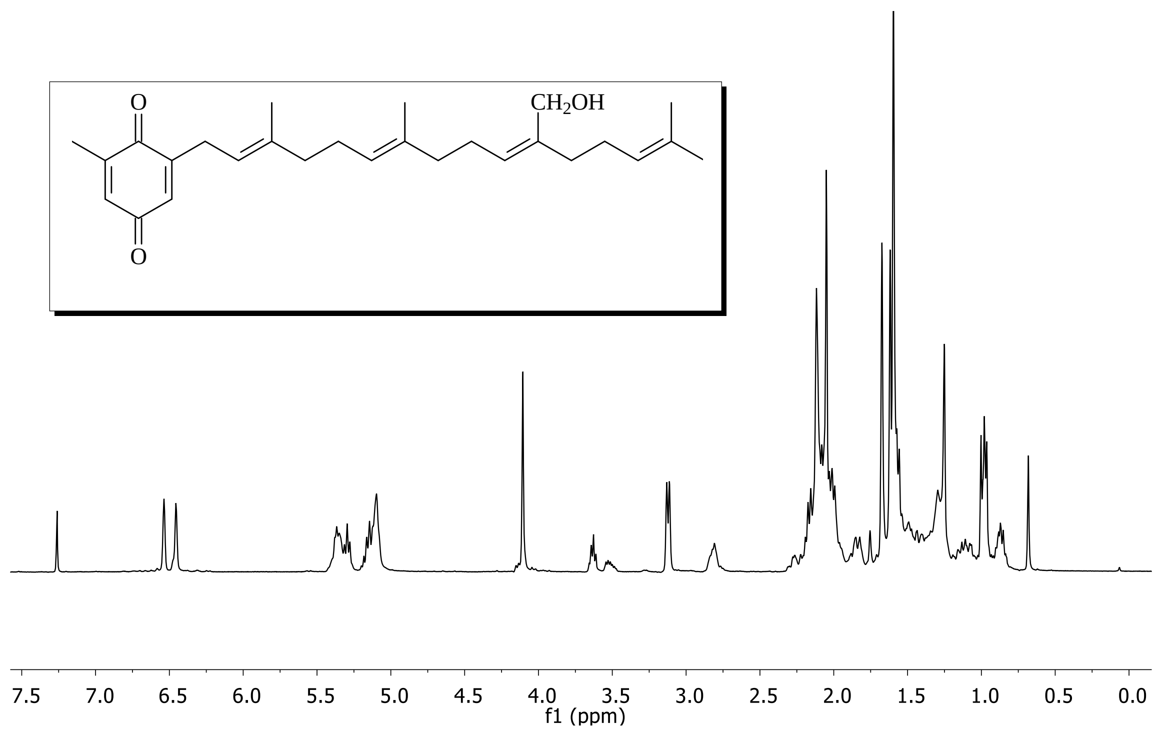

Figure S19. ^{1}H NMR spectrum of sargaquinol (**6**) (400 MHz, $CDCl_3$)

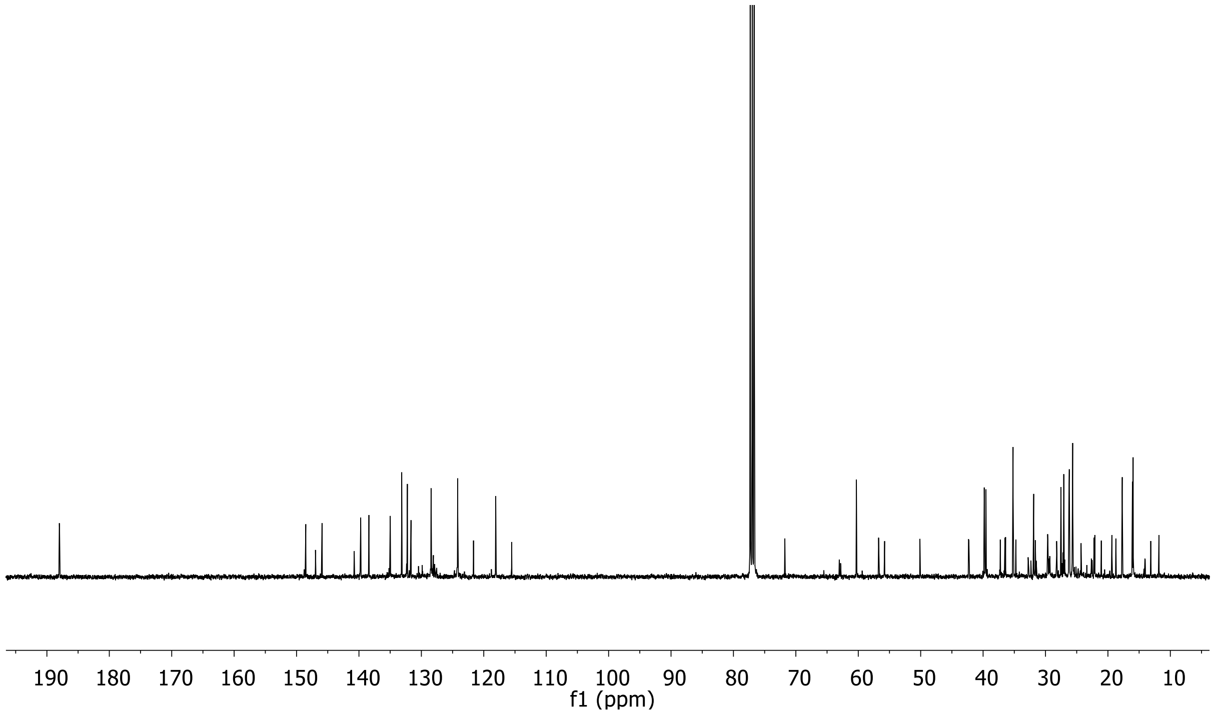

Figure S20. ^{13}C NMR spectrum of sargaquinol (**6**) (100 MHz, $CDCl_3$)

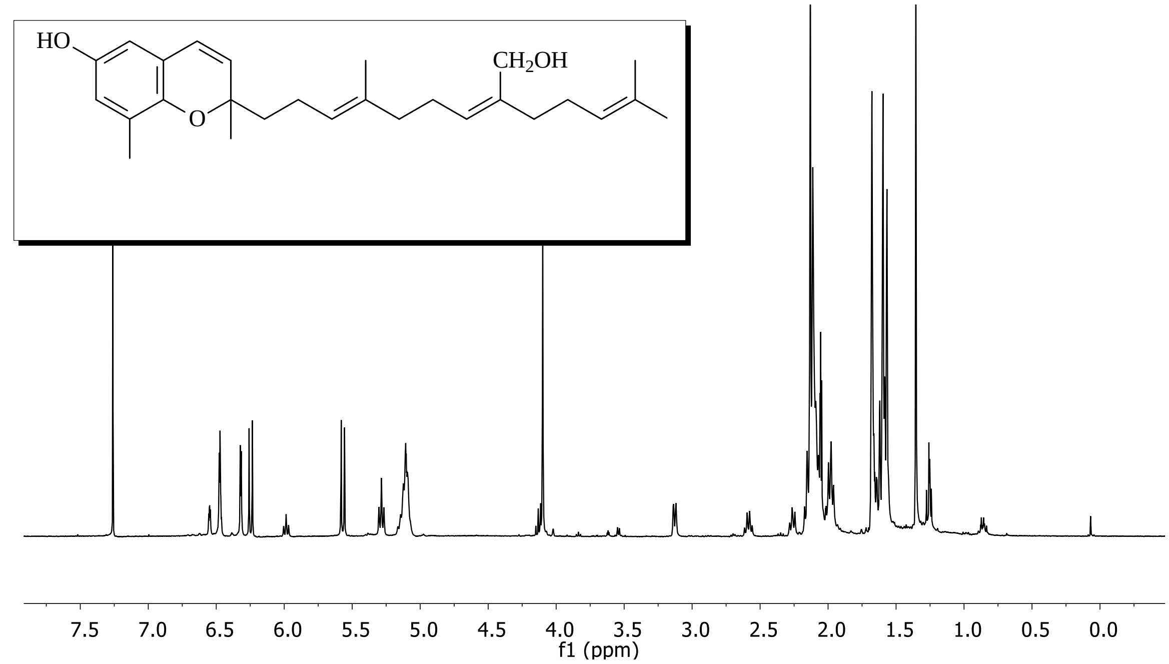

Figure S21. ^{1}H NMR spectrum of sargachromendiol (**8**) (400 MHz, $CDCl_3$)

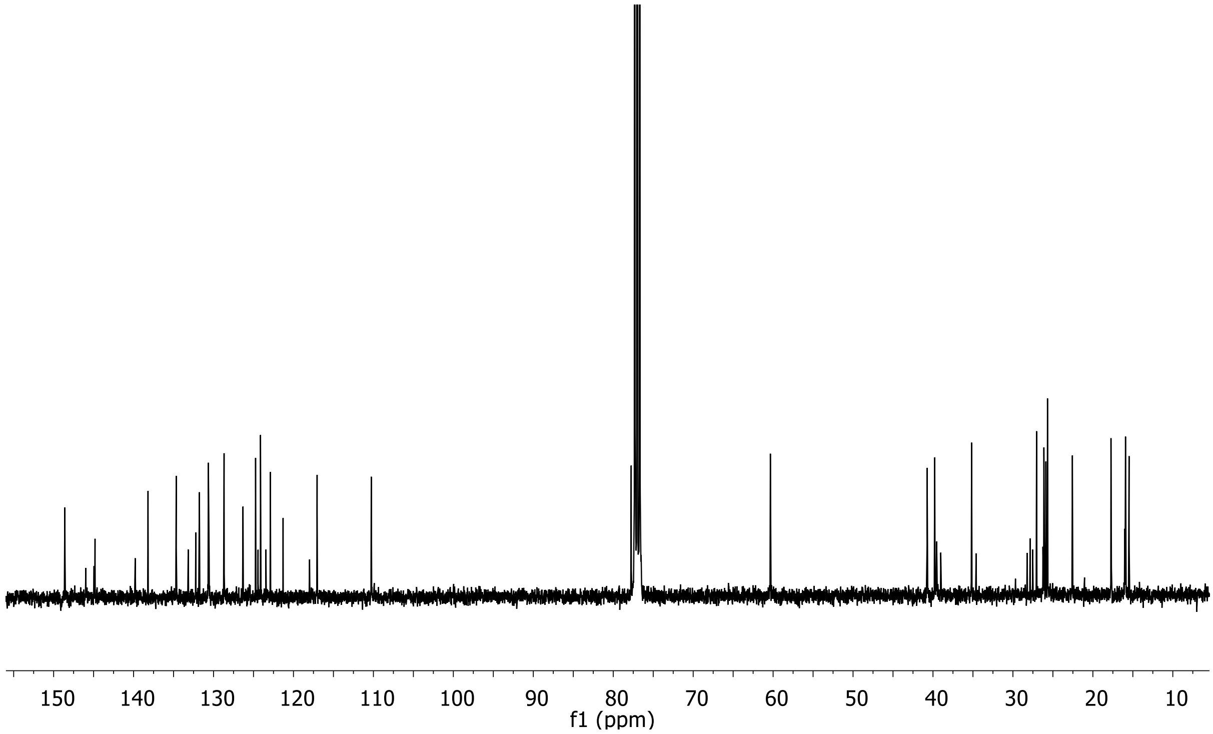

Figure S22. ^{13}C NMR spectrum of sargachromendiol (**8**) (100 MHz, $CDCl_3$)

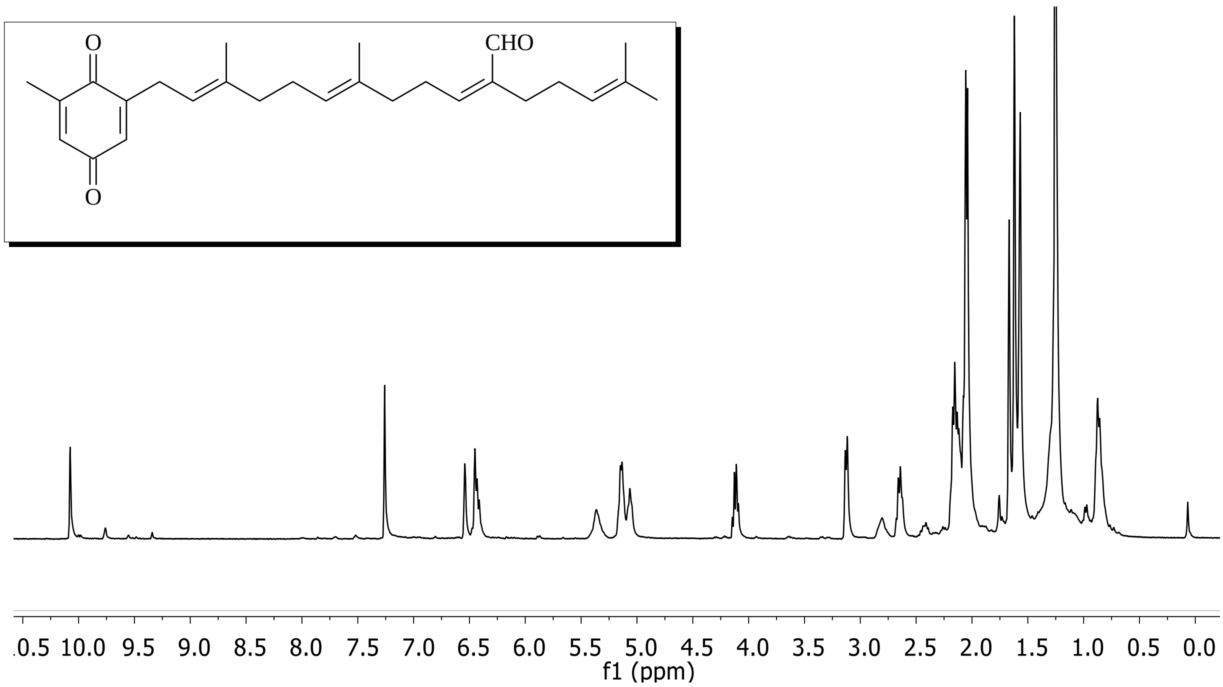

Figure S23. ^{1}H NMR spectrum of 10′Z-sargaquinal (**4**) (600 MHz, $CDCl_3$)

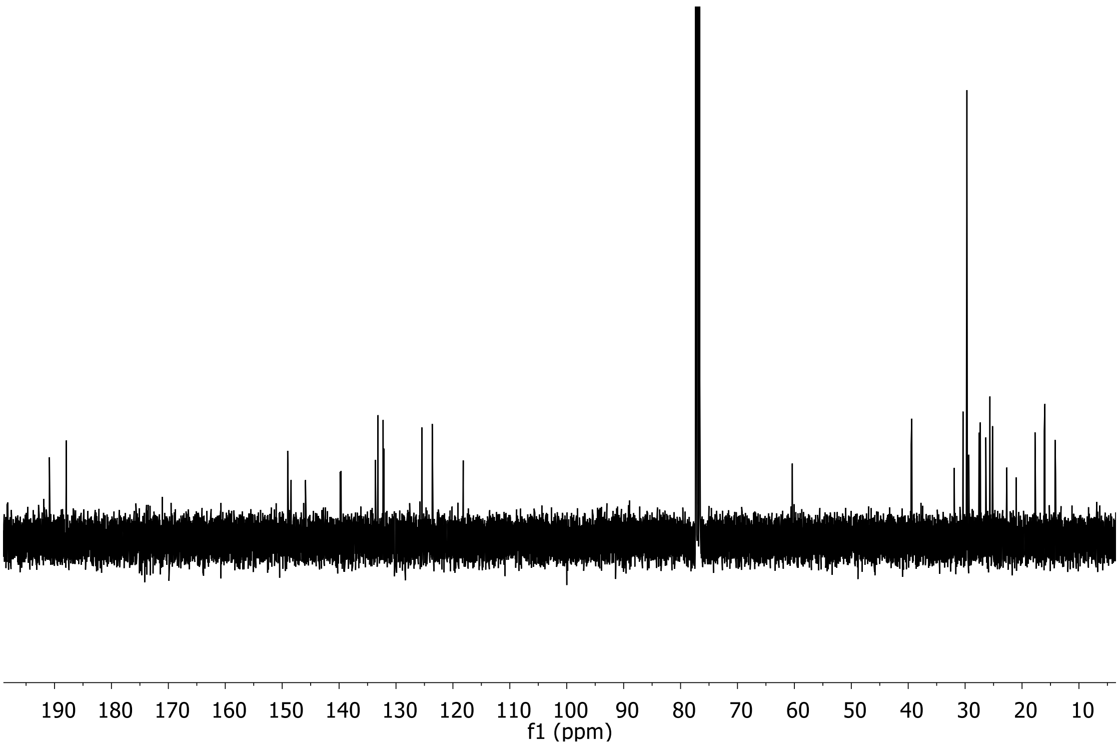

Figure S24. ^{13}C NMR spectrum of 10′Z-sargaquinal (**4**) (100 MHz, $CDCl_3$)
